# Correction: Changes in Soil Physical and Chemical Properties in Long Term Improved Natural and Traditional Agroforestry Management Systems of Cacao Genotypes in Peruvian Amazon

**DOI:** 10.1371/journal.pone.0136784

**Published:** 2015-08-21

**Authors:** The PLOS ONE Staff

## Notice of Republication

This article was republished on August 11, 2015, to correct an error in the title: the word “Term” was changed to “Lerm” during the production process. The publisher apologizes for the error. Please download this article again to view the correct version. The originally published, uncorrected article and the republished, corrected article are provided here for reference.

## Supporting Information

S1 File Originally published, uncorrected article. (PDF)

S2 File Republished, corrected article. (PDF)
