# Supplementary material for: Correction: Changes in Soil Physical and Chemical Properties in Long Term Improved Natural and Traditional Agroforestry Management Systems of Cacao Genotypes in Peruvian Amazon
Source: PLoS One. 2015 Aug 21;10(8):e0136784. doi: 10.1371/journal.pone.0136784 (PMC4546628; doi:10.1371/journal.pone.0136784)
Supplement: S1 File — (PDF) [file pone.0136784.s001.pdf]

PLOS ONE

RESEARCH ARTICLE

Changes in Soil Physical and Chemical Properties in Long Lerm Improved Natural and Traditional Agroforestry Management Systems of Cacao Genotypes in Peruvian Amazon

Enrique Arévalo-Gardini[1] *, Manuel Canto[2], Julio Alegre[2], Oscar Loli[2], Alberto Julca[2], Virupax Baligar[3]

1 Instituto de Cultivos Tropicales, (ICT), Tarapoto, Perú, **2** Universidad Nacional Agraria La Molina (UNALM), Lima, Perú, **3** U.S. Department of Agriculture/Agricultural Research Service, Beltsville Agricultural Research Center, Beltsville, Maryland, United States of America

* enriquearevaloga@gmail.com

CrossMark
click for updates

OPEN ACCESS

Citation: Arévalo-Gardini E, Canto M, Alegre J, Loli O, Julca A, Baligar V (2015) Changes in Soil Physical and Chemical Properties in Long Lerm Improved Natural and Traditional Agroforestry Management Systems of Cacao Genotypes in Peruvian Amazon. PLoS ONE 10(7): e0132147. doi:10.1371/journal.pone.0132147

Editor: Raffaella Balestrini, Institute for Sustainable Plant Protection, C.N.R., ITALY

Received: July 16, 2014

Accepted: June 10, 2015

Published: July 16, 2015

Copyright: This is an open access article, free of all copyright, and may be freely reproduced, distributed, transmitted, modified, built upon, or otherwise used by anyone for any lawful purpose. The work is made available under the Creative Commons CC0 public domain dedication.

Data Availability Statement: ICT (Instituto De Cultivos Tropicales) legal guidelines prohibit uploading of experimental data of research carried under ICT authority on a stable, public repository such as Dryad or Figshare, I attach the average of data as a Supporting Information file. However on case by case basis ICT could share selected experimental data with interested readers and request for specific data should be made to the following individual of ICT. Dr. Enrique AREVALO—GARDINI" enriquearevaloga@gmail.com Av Cerro

Abstract

Growing cacao (*Theobroma cacao* L.) in an agroforestry system generates a productive use of the land, preserves the best conditions for physical, chemical and biological properties of tropical soils, and plays an important role in improving cacao production and fertility of degraded tropical soils. The aim of this study was to evaluate the impact of two long term agroforestry systems of cacao management on soil physical and chemical properties in an area originally inhabited by 30 years old native secondary forest (SF). The two agroforestry systems adapted were: improved natural agroforestry system (INAS) where trees without economic value were selectively removed to provide 50% shade and improved traditional agroforestry system (ITAS) where all native trees were cut and burnt in the location. For evaluation of the changes of soil physical and chemical properties with time due to the imposed cacao management systems, plots of 10 cacao genotypes (ICS95, UF613, CCN51, ICT1112, ICT1026, ICT2162, ICT2171, ICT2142, H35, U30) and one plot with a spontaneous hybrid were selected. Soil samples were taken at 0-20, 20-40 and 40-60 cm depths before the installation of the management systems (2004), and then followed at two years intervals. Bulk density, porosity, field capacity and wilting point varied significantly during the years of assessment in the different soil depths and under the systems assessed. Soil pH, CEC, exchangeable Mg and sum of the bases were higher in the INAS than the ITAS. In both systems, SOM, Ext. P, K and Fe, exch. K, Mg and Al+H decreased with years of cultivation; these changes were more evident in the 0-20 cm soil depth. Overall improvement of SOM and soil nutrient status was much higher in the ITAS than INAS. The levels of physical and chemical properties of soil under cacao genotypes showed a marked difference in both systems.

Escalera Mz."C" Lote1, Urb. Las Praderas, Banda del Shilcayo Tarapoto, San Martín, Perú Tel/Fax 51-42-522361

Funding: This study was supported by Instituto de Cultivos Tropicales (ICT), United States-Department of Agriculture—Agricultural Research Service (USDA-ARS) and the United States Embassy (INL) in Lima-Peru.

Competing Interests: The authors have declared that no competing interests exist.

Introduction

Cacao is one of the most important perennial crops of the Peruvian tropics and Peru is the third largest producer of organic cacao in the world [1]. In the last 10 years, the cultivated area under cacao in Peru has increased at the rate of 4,800 ha yr^{-1} [2]. On acid soils of the Amazon region, traditional cacao planting is preceded by cutting and selling trees of economic significance then burning the remaining aboveground biomass on the location. In some areas cacao is planted in thinned secondary forest (SF) and the remaining slashed vegetation is used as mulch. Such types of shifting cultivations have drastically changed climate factors and ecological patterns due to removal of the natural forest [3, 4]. These methods of land management often lead to loss of soil and nutrients; and consequently, affect the dynamic patterns of biogeochemical cycles.

The consequences of deforestation in the Amazon region are evident in the deterioration of natural resources with a loss of biodiversity, productive capacity of the soil and its consequential surrender to the natural regeneration of vegetation as compared to natural forest [5]. In addition, poor agricultural practices degrade the forest ecosystem, mainly the soil [6], which is a complex ecosystem bound by physical-chemical parameters [7]. Success of sustainable production systems in the tropical areas is dependent on the proper management of the physical and chemical properties of these soils [8]. One way to mitigate these deforestation practices in the Peruvian jungle is to provide viable alternatives such as agroforestry systems of crop management for farmers who practice slash and burn crop production thereby accelerating deforestation and soil degradation and increasing rural poverty [9]. Archaeological evidence and historical accounts of the Inca indicate that Peru has a long history of agroforestry [10, 11].

The transition from a traditional farming system to a low external input sustainable system is accompanied by a set of changes in soil chemical properties and processes that affect soil fertility [6]. Traditional agricultural systems have led to a continuing degradation of soil resources, particularly from the chemical point of view, resulting in a loss of agricultural productivity reflected in lower yields and higher environmental problems [12].

The adoption of some typical farming practices for sustainable crop production include avoiding of cutting and burning or removing of the native vegetation, use of SF trees as temporary and permanent shade, use of cover crops and reducing the use of synthetic fertilizers and pesticides, which cause fundamental differences in the quantitative and qualitative flow of soil nutrients. These changes affect the availability of nutrients for growing crops either directly by contributing to the availability of nutrients or indirectly by influencing the physical and chemical environment of the soil [13]. Agroforestry systems integrate trees with agricultural crops [14] such as cacao or coffee for example [11, 15], and have the potential to enhance soil fertility, maintain the soil organic matter status, promote efficient nutrient cycling, reduce erosion, improve water quality, enhance biodiversity, increase a esthetics [14, 16], and over the long-term are also sustainable and appropriate for soil fertility conservation and improve soil health, and sequester carbon [16, 17, 18, 19, 20].

In recent years the cultivation of cacao has enjoyed growing acceptance as a more profitable crop in the Peruvian jungle, and this has led to accelerated forest conversion with the same impacts as have happened in other parts of the world [6]. Most current cacao plantations depend on a traditional system of clearing areas for planting of crops such as maize, beans, and bananas and finally planting of cacao [3, 4].

On the other hand, efforts are in progress in the region to promote sustainable alternative systems of farming in which vegetation under the trees is cut but leaving some of the forest trees for shade, which are subsequently replaced by higher-yielding and high value tree species [3]. This is a way to curb the negative impact of the logging and burning of native trees, thereby

attempting to maintain a balance in a manner similar to that of a primary forest, contributing to the conservation of the physical-chemical properties of the soil and the flora and fauna that inhabit these systems [6]. Therefore, agroforestry systems of crop cultivation play an important role in improving nutrient flow and soil quality [21].

Soils managed in sustainable and conventional farming systems with organic practices have shown high levels of organic matter (SOM) and total nitrogen [22]. The increase of SOM in soil after implementing a sustainable farming system occurs slowly and usually detecting these differences takes several years [23]. The changes in other soil properties are more variable, perhaps due to differences in climate, soil type, crops grown, and duration of culture system implemented [24]. Because these soil properties are critical in determining the fertility of agricultural soils, the ability to predict and manage their dynamics and intensity in time and space will facilitate the transition to a sustainable model with low dependence on external inputs.

This research evaluates the changes that occurred in the soil physical and chemical properties in the long-term improved natural agroforestry system (INAS) and the improved traditional agroforestry system (ITAS) planted with 11 cacao genotypes in the Peruvian Amazon.

Methods

Location and installation of the experiment

This study was conducted in the Experimental Farm "El Choclino" of property of the Instituto de Cultivos Tropicales (ICT); its administration provided all necessaries facilities and permits to conduct this long term study. The ICT is located in the Department of San Martin, Province of San Martin, and District of La Banda de Shilcayo. (Fig 1). Geographically, the experimental site is located at 6° 28' 37.3" S 76° 19' 54.6" W, at an altitude between 500 and 530 m.a.s.l. In accordance with the Peruvian ecological map, the study site is located within the living area of Pre-Mountain Tropical Dry Forest (BSPmT) [25]. The terrain is steep in some areas with slopes greater than 50%, but mostly dominated by slopes of less than 50%. The primary vegetation was SF native to the region of approximately 30 years old, where there were forest species

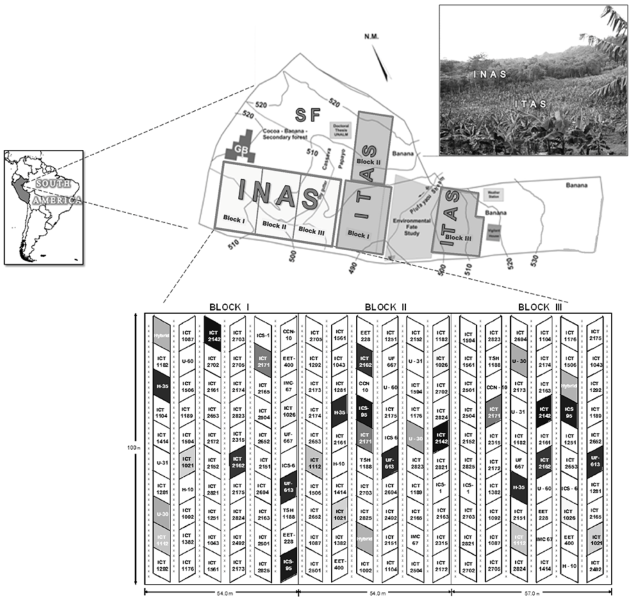

Fig 1. Map Showing the Location of Cacao Agroforestry Management Field Study (INAS, ITAS).

doi:10.1371/journal.pone.0132147.g001

Table 1. Physical and Chemical Properties of Soil Under SF, Before the Initiation (2004) of Cacao Agroforestry Management Systems (INAS, ITAS).

Soil properties		Depth		
	Unit	0–20 cm	20–40 cm	40–60 cm
		**Physical Properties**		
Sand	%	44	38.00	35.00
Clay	%	29	38.00	45.00
Silt	%	27	24.00	20.00
Texture		Clay loam	Clay loam	Clay
Bulk density	g cm^{-3}	1.39	1.44	1.42
Porosity	%	47.75	45.65	46.40
Field Capacity	%	31.40	35.55	39.30
Wilting point	%	18.05	23.20	27.00
Plant Av. Water	%	13.35	12.35	12.30
		**Chemical Properties**		
pH	(1:1)	5.65	5.35	4.85
EC	dS m^{-1}	0.43	0.38	0.44
SOM	%	3.55	1.60	1.10
P Ext.	μg g^{-1}	6.50	4.10	3.30
K Ext.	μg g^{-1}	107.50	76.50	79.50
Fe Ext.	μg g^{-1}	123.05	224.15	70.00
Cu Ext.	μg g^{-1}	1.25	1.65	1.15
Zn Ext.	μg g^{-1}	1.80	2.10	1.40
Mn Ext.	μg g^{-1}	10.95	10.35	6.40
K Exch.	cmol kg^{-1}	0.21	0.17	0.16
Ca Exch.	cmol kg^{-1}	17.38	15.71	17.82
Mg Exch.	cmol kg^{-1}	1.94	1.26	1.03
Al+H Exch.	cmol kg^{-1}	0.30	1.30	2.50
CEC	cmol kg^{-1}	19.82	18.43	21.51

doi:10.1371/journal.pone.0132147.t001

(58%), palm (3%), and lianas and herbaceous (39%) [Personnel information]. In the experimental area, the average annual rainfall is around 1250 mm with an average temperature of 26°C and an average relative humidity of 87%. The predominant soil under the experiment is Order: Alfisol [26], the surface (0–20 cm) texture is clay loam, pH of 5.65, with a soil organic matter (SOM) of 3.55%. Details of SF soil properties at various depths are given in Table 1.

In 2004 land preparation commenced to install two long term cacao management treatments: Improved Native Agroforestry System (INAS) and an Improved Traditional Agroforestry System (ITAS) and is expected to run for a period of 25 years. In the INAS cacao management system, cacao genotypes were established under thinned native forest, whereas in the ITAS cacao management system cacao genotypes were established on land cleared by the slash and burn method. In INAS, weeds and shrubs were removed manually and the native tree density was selectively reduced by cutting un-economical trees to achieve approximately 50% shade. In ITAS trees, brush and other vegetation were removed by following the slash and burn method of the local cacao farmers. In this system, all the weeds, shrubs and SF trees were manually cut and allowed to dry then burnt on location (June, 2004). Yellow corn (*Zea mays*) var. Marginal T-28 was planted at a spacing of 0.4 x 0.8 m (September, 2004) and at the same time different varieties of banana (*Musa sp*) such as Inguiri, bellaco plantain and Isla banana

Table 2. Characteristics and Management Practices Adapted for Cacao Agroforestry Management Systems (INAS, ITAS) for 2004 to 2010 Period.

Practices	System	
	Improved Native Agroforestry System INAS	Improved Traditional Agroforestry System—ITAS
**Primary vegetation**	Secondary forest (SF) native to the region of approximately 30 years old, where there were forest species (58%), palm (3%), and lianas and herbaceous (39%)	Secondary forest native to the region of approximately 30 years old, where there were forest species (58%), palm (3%), and lianas and herbaceous (39%)
**Management System Installation**	Under thinned native forest. weeds and shrubs were removed manually and the native tree density was selectively reduced by cutting un-economical trees to achieve approximately 50% shade.	Trees, brush and other vegetation were removed by following the slash and burn method of the local cacao farmers. In this system, all the weeds, shrubs and SF trees were manually cut and allowed to dry then burnt on location
**Intercropping**	None, but when the shade was less than 50%, banana (*Musa* sp) was planted to increase the shade in some areas	Yellow corn (*Zea mays*) var. Marginal T-28 was planted at a spacing of 0.4 x 0.8 m (September, 2004) and at the same time different varieties of banana (*Musa sp*) such as Inguiri, bellaco plantain and Isla banana (*Musa ensete*) were planted at a spacing of 4 x 3 m to provide early shade to young cacao. In March 2005, var. Huascaporoto bean (*Phaseolus vulgaris*) was seeded as a second annual crop
**Additional economical shade trees**	Shimbillo (*Inga* sp), Pashaco (*Macrolobium acaciafolium*), Capirona (*Calycophyllum spruceanum*), Tornillo (*Cedrelinga cateaniformes*), and Paliperro (*Vitex pseudolia*) at a spacing of 8 x 9 m to provide about 138 trees ha^{-1} of each specie	Shimbillo (*Inga* sp), Pashaco (*Macrolobium acaciafolium*), Capirona (*Calycophyllum spruceanum*), Tornillo (*Cedrelinga cateaniformes*), and Paliperro (*Vitex pseudolia*) at a spacing of 8 x 9 m to provide about 138 trees ha^{-1} of each specie
**Cacao root stocks**	IMC-67	IMC-67
**Fertilization (at planting)**	250 g seabirds manure, mixed with inorganic fertilizer 0.5 kg (14N-12P-4K) was added to each pit	250 g seabirds manure, mixed with inorganic fertilizer 0.5 kg (14N-12P-4K) was added to each pit
**Fertilization (during grow)**	Mineral fertilization 0.5 kg of 196 N -250P-220K (urea, DAP, PCl) per plant at 2010	Mineral fertilization 0.5 141N-181P-109K (urea, DAP, PCl) per plant at 2010
**Weed control as needed**	Hand weeding as needed	Hand weeding as needed
**Diseases and Insect control**	IPM[1]	IPM[1]
**General pruning**	One time per year (September to October)	One time per year (September to October)

[1]IPM are referred to integrated pest management that consist in the optimization of cultural practices [15], such as removal of sick and diseases infested parts of the tree, adequate pruning,and protection of pods with cooper fungicide in the period of pod development (four times per year between December to March). DAP: Diammonium phosphate, PCl: Potassium chloride.

doi:10.1371/journal.pone.0132147.t002

(*Musa ensete*) were planted at a spacing of 4 x 3 m to provide early shade to young cacao. In March 2005, var. Huascaporoto bean (*Phaseolus vulgaris*) was seeded as a second annual crop.

In order to plant cacao root stocks in both the systems, pits of 0.3 x 0.3 x 0.3 m size were dug at spacing of 2 m within plants by 3 m between rows, to achieve 1768 plants ha^{-1}. At the time of planting, 250 g of guano (seabirds manure) mixed with inorganic fertilizer 0.5 kg (14N-12P-4K) was added to each pit. Four months old seedlings of cacao rootstock IMC- 67 (strong and deep rooted, resistant to *Ceratocystis fimbriata* disease) were planted in April-May 2005. Additional shade trees were planted in both systems during November 2005 composed of native species of the area such as Shimbillo (*Inga sp*), Pashaco (*Macrolobium acaciafolium)*, Capirona (*Calycophyllum spruceanum)*, Tornillo (*Cedrelinga cateaniformes)*, and Paliperro (*Vitex pseudolia)* at a spacing of 8 x 9 m to provide about 138 trees ha^{-1} of each species. In both management systems, weeds were removed manually by machete. After planting the cacao rootstock seedlings in both systems of management, areas of 60 m^2 were delineated as a single plot, to accommodate 10 plants of each genotype. Well-developed disease-free shoot cuttings from selected cacao genotypes were side grafted onto six month old rootstocks (October-November 2005), more detailed of activities is summarized in the Table 2, Fig 1 and Fig 2.

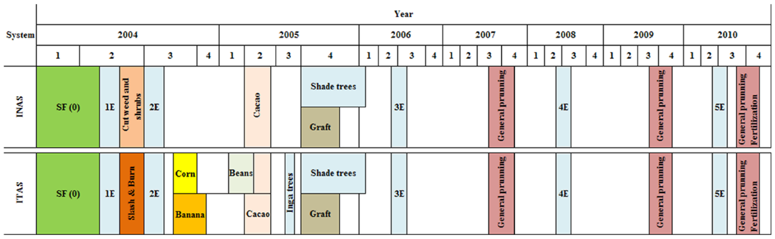

Fig 2. Management practices adapted and sequence of crops adapted in cacao agroforestry systems. SF (0) land under secondary forest before the implementation of cacao, 2004. E = soil sampling, spaces in blank indicates maintenance of cacao.

doi:10.1371/journal.pone.0132147.g002

In each block there were 60 experimental plots to accommodate 60 accessions (59 improved native and international genotypes and one non-grafted spontaneous native hybrid) and each one of them was assigned to a plot. For both the systems of management a random block design was adapted and experimental units (genotypes) were replicated in three blocks (5,500 m^2 each). In both the systems of management, 11 representative cacao accessions [three international genotypes (ICS-95, UF-613, CCN-51), seven native genotypes [(ICT-1112, ICT-1026, ICT-2162, ICT-2171, ICT-2142, H-35, U-30) and one spontaneous hybrid] were selected in each block for detailed evaluation of changes in soil physical and chemical properties due to different long term agroforestry cacao management systems (Table 3).

Soil sampling

Soil samples were collected from three depths (0–20 cm, 20–40 cm, 40–60 cm) during 2004 just prior to the installation of the cacao management studies and designated as soil samples under secondary forest (SF) samples (Table 1). At the time of installations additional soil samples were collected for each depth, from both the systems of cacao management and designated as 2004 initial soil samples. For ITAS system soil samples were collected two weeks after burn of the native forest. In each cacao accessions plot, 10 soil samples were taken randomly throughout the plot in zigzag type direction. Litter was removed before soil sampling. A stainless steel tube with 2 cm diameter and 80 cm in length was driven at each sampling site to the

Table 3. Cacao Genotypes and Spontaneous Hybrid Selected for Evaluation of their Impact on Soil Physical and Chemical Properties under Long Term Cacao Agroforestry Management Systems (ITAS, INAS).

Genotype	Description	Origin	Characteristics *
**ICS 95**	Imperial College Selection	Trinidad	SC, FTRR,
**UF 613**	United Fruit Series	Costa Rica	SC,
**CCN 51**	Castro Naranjal Collection	Ecuador	SC, WbR, HP
**ICT 1112**	Tropical Crops Institute	Juanjui–Peru	SC
**ICT 1026**	Tropical Crops Institute	Juanjui–Peru	SC
**ICT 2162**	Tropical Crops Institute	Tocache–Peru	SC
**ICT 2171**	Tropical Crops Institute	Tocache–Peru	SC, WbR,
**ICT 2142**	Tropical Crops Institute	Tocache–Peru	SC, WbR
**H 35**	Huallaga Collection	Huallaga- River Basin- Peru	SC
**U 30**	Ucayali Collection	Ucayali- River Basin-Peru	SC
**Spontaneous hybrid**	Hybrid (Control)	Peru	SI

*SC = Self-compatible, SI = Self-incompatible, WbR = Witches' Broom Resistant, HP = High productivity, FTRR = Frosty Pod Rot Resistant.

doi:10.1371/journal.pone.0132147.t003

desired depth to obtain a soil sample and these were mixed thoroughly and a 1 kg of composite sample was transported to the lab, air dried, ground and passed through a 2 mm sieve and stored at room temperature. In both systems and in all the plots under the 11 cacao accessions soil sampling from three desired depths, similar to initial sampling was repeated during 2006, 2008 and 2010.

Determination of soil physical and chemical properties

The initial analysis of the physical and chemical properties of soil samples was done at the soil and plant analytical lab of the National Agrarian University of La Molina (UNALM, Universidad Nacional Agraria La Molina, Lima and soil samples collected during the subsequent years were analyzed at the laboratory of soil and water of the Tropical Crops Institute (ICT, Instituto de Cultivos Tropicales), Tarapoto. Soil analyses in both of these labs followed similar protocols recommended by Anderson and Ingram [27]. Soil texture was determined with Bouyoucos densimeter after shaking the soil vigorously with NaOH (1 mol L^{-1}) as a dispersant; bulk density (BD) was measured by the cylinder method and from the BD porosity was computed [(1-BD/2.65) x 100] [27]. Soil water content at field capacity (FC), wilting point (WP) and plant available water (PAW) were estimated based on texture and organic matter content according to the model proposed by Saxton and Rawls [28]. The chemical properties determined were: pH (1:1 H_2O) by the potentiometric method, electrical conductivity (EC) by conductivity meter, extractable ions (Ext. P, K, Fe, Cu, Zn, Mn) by Olsen modified method [27], exchangeable bases (Exch. K, Ca, Mg) for soils with pH $\leq$ 5.5 by 1 M ammonium acetate and for soils with pH $>$ 5.5 by1 N KCl [24], exchangeable acidity (Exch. Al+H) by the Yuan method [27], and soil organic matter (SOM) by the Walkley and Black method [27]. Ca, Mg, K, Fe, Cu, Mn, and Zn in the extractants were determined by atomic absorption spectrophotometry, P in the extractant by use of the ascorbic-Molybdate color development method and detected by colorimetry [27]. CEC (Cation Exchange Capacity) was calculated as the sum of exchangeable bases (Exch, K, Ca, Mg) plus exchangeable Al+H [27].

Statistical Analysis

All statistical analysis was carried out using InfoStat, 2013 version [29]. Data of physical and chemical soil properties were analyzed separately by depth (0-20cm, 20-40cm, 40-60cm) with linear mixed effect models with repeated measures using the function “lme” from the package “nlme” [30, 31, 32] of the statistical software R version 3.1.1 [33]. We used a model with *System (S)*: INAS, ITAS, *Year (Y)*:2004, 2006, 2008 and 2010 and the interaction of *S x Y* as a fixed effects and *Block* (n = 3) and *Genopytes (G)* as random intercepts. In the model of repeated measures, the experimental units are considering a random factor and the time (*Y*) as fixed effect [31, 32]. The fixed effect *Year* was included in the model to account for repeated measures on the same *System* for 2004, 2006, 2008 and 2010, mean comparisons were made by DGC (alpha test = 0.05) [31]. This effect indicates how the dynamic of physical and chemical soil properties is across time frame of the study (Figs 3, 4, 5 and 6). For the effect of *Genotype*, we used also the linear mixed effect models, the model was *System* (n = 2), *Genotype* (n = 11) and the interaction *S x G* as a fixed effects, the *Blocks* (n = 3) as random intercepts the analysis was performed at the end of the experiment (2010) and at 0-20cm depth and compared with the SF and the time of study installation. Mean comparisons were made by DGC (alpha test = 0.05) [31]

Results and Discussion

Effects of INAS and ITAS cacao management on soil physical properties

The soil physical properties examined in INAS and ITAS over the six year period (2004 to 2010) were: soil bulk density (BD), porosity (Po), wilting point (WP), field capacity (FC) and plant available water (PAW). The average values for these properties is showed in S1 Table and values with statistical significance are shown in Table 4. Values for these physical properties for SF are given in Table 1. Over all changes in physical properties at varying soil depths of two management systems at different time frames and values for natural forest irrespective of genotypes are give in Fig 3.

During the years of assessment, overall, the cacao management systems significantly influenced soil physical properties such as BD and porosity at all soil depths; FC and WP at depths of 0-20cm and 20-40cm; and PAW at soil depth of 20-40cm and 40-60cm, as compared with the soil physical properties of the SF (Table 4).

Bulk density (BD). The BD values at the start of experiment in the SF top layer were lower than deeper layers (1.39 g cm^{-3}, 1.42 g cm^{-3} respectively) (Table 1).Overall the BD in ITAS was significantly higher than INAS during the years and soil depths assessed (Fig 3). The BD values were inversely related to soil porosity as high BD resulted in lower total soil porosity

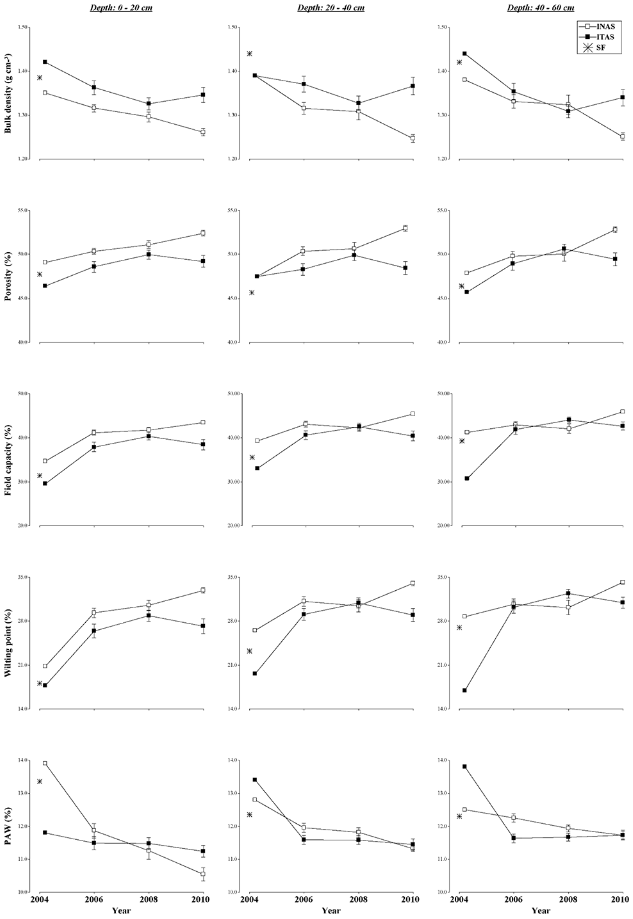

Fig 3. Changes in soil physical properties. Under long term agroforestry management systems (INAS, ITAS) as compared to soil properties of SF at different depths (0–20, 20–40, 40–60 cm) during 2004 to 2010 experimental period.

doi:10.1371/journal.pone.0132147.g003

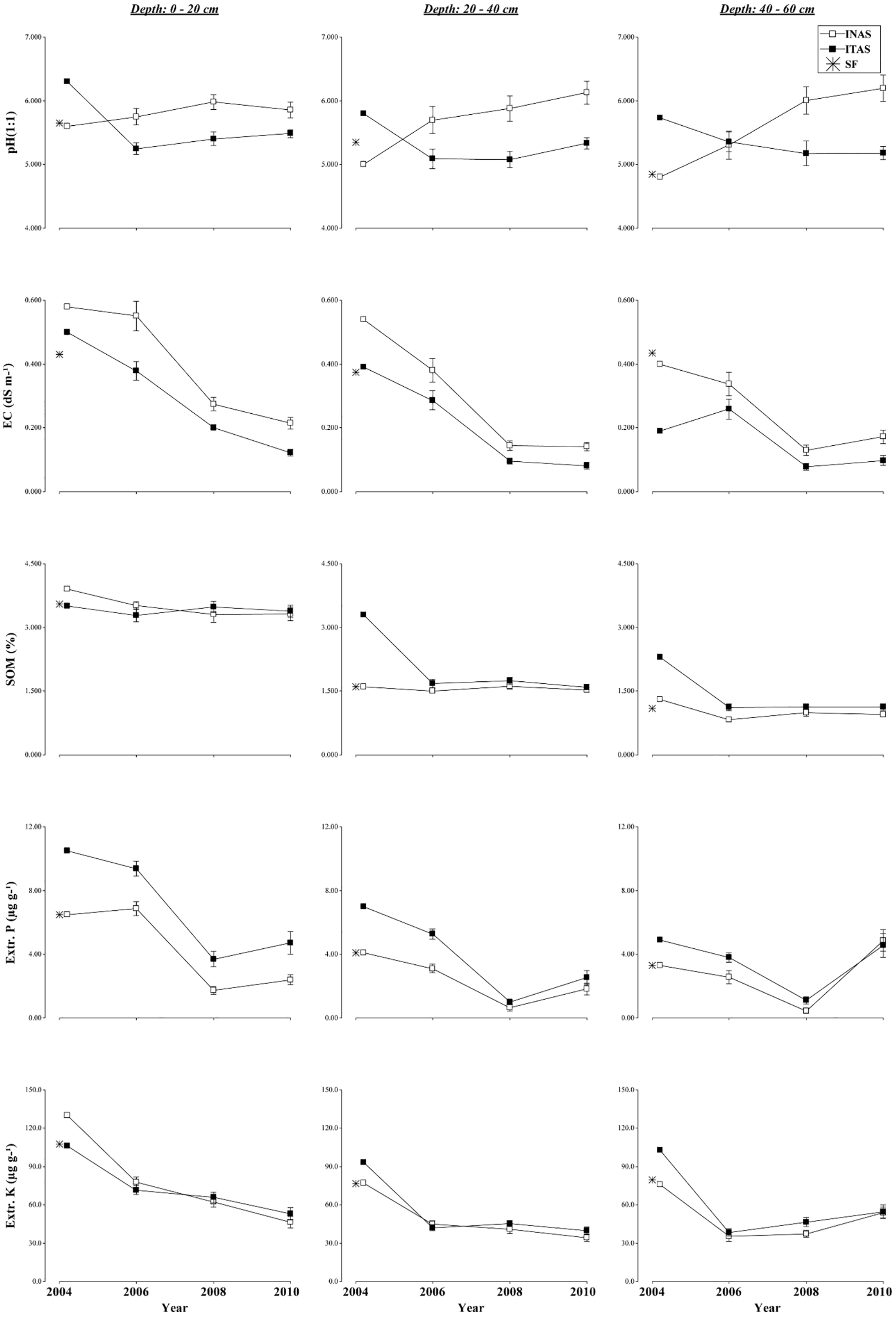
Depth: 0 - 20 cm
Depth: 20 - 40 cm
Depth: 40 - 60 cm
INAS
ITAS
SF
pH(1:1)
EC (dS m-1)
SOM (%)
Extr. P (µg g-1)
Extr. K (µg g-1)
2004
2006
2008
2010
Year

Fig 4. Changes in soil chemical properties. Under long term agroforestry management systems (INAS, ITAS) as compared to soil properties of SF at different depths (0–20, 20–40, 40–60 cm) during 2004 to 2010 experimental period.

doi:10.1371/journal.pone.0132147.g004

[34]. The lower BD under INAS can have a positive effect on the development of roots, especially in tree plantations because when soil bulk density increases, soil strength increases and soil aeration decreases, leading to adverse effects on root growth [35]. The variation in BD is evident in the surface soil layer, while in the deeper soil layers, it tends to be the same in both systems. BD on an average were less in surface soil layers and increased with increasing soil depth, these results are similar to the report by Perrin et al [36] in a study of conversion of forest to agriculture in Amazonia with the chop-and-mulch method. Production systems that conserve soil, such as INAS, continuously introduce fresh organic matter, which was essential to maintaining a good soil structure [37]. Variations in the soil physical properties observed in these two systems of management are attributable to a low soil disturbance in INAS coupled with mulching effects of frequent additions of organic matter through litter fall. Vegetative tree cover in INAS reduced soil degradation by reducing the impact of rain drops and abrupt changes in the relative humidity. Fresh organic matter frequently added is an ideal substrate for microbial activity, which acts as an agent for improving the stability of the aggregates and promotes better pore distribution and because decomposing organic matter components are less dense than the mineral components that leads to lowering of surface soil BD [37, 38].

Moreover, accumulation of plant litter produced by the various trees and cacao allows greater infiltration of rainwater thereby preventing the quick loss of soil moisture and increasing the soil water holding capacity. This shows that soil physical properties are altered by the

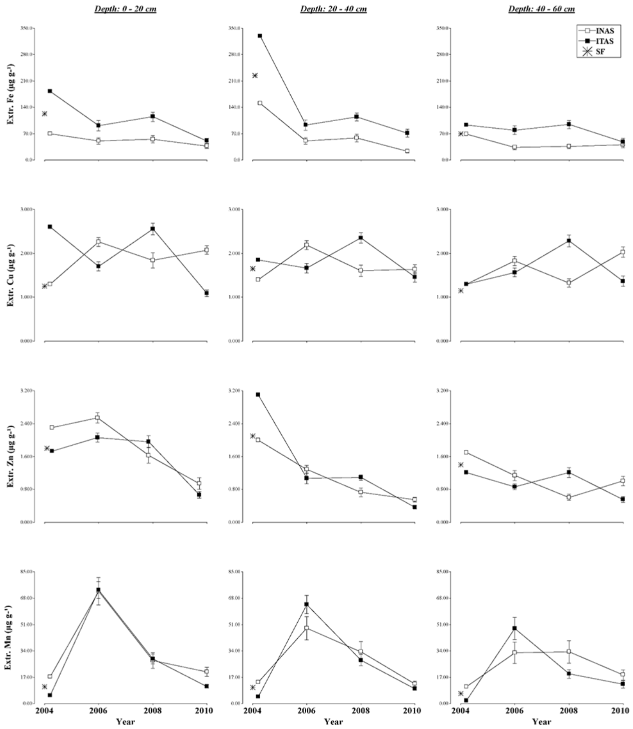

Fig 5. Changes in soil chemical properties. Under long term agroforestry management systems (INAS, ITAS) as compared to soil properties of SF at different depths (0–20, 20–40, 40–60 cm) during 2004 to 2010 experimental period.

doi:10.1371/journal.pone.0132147.g005

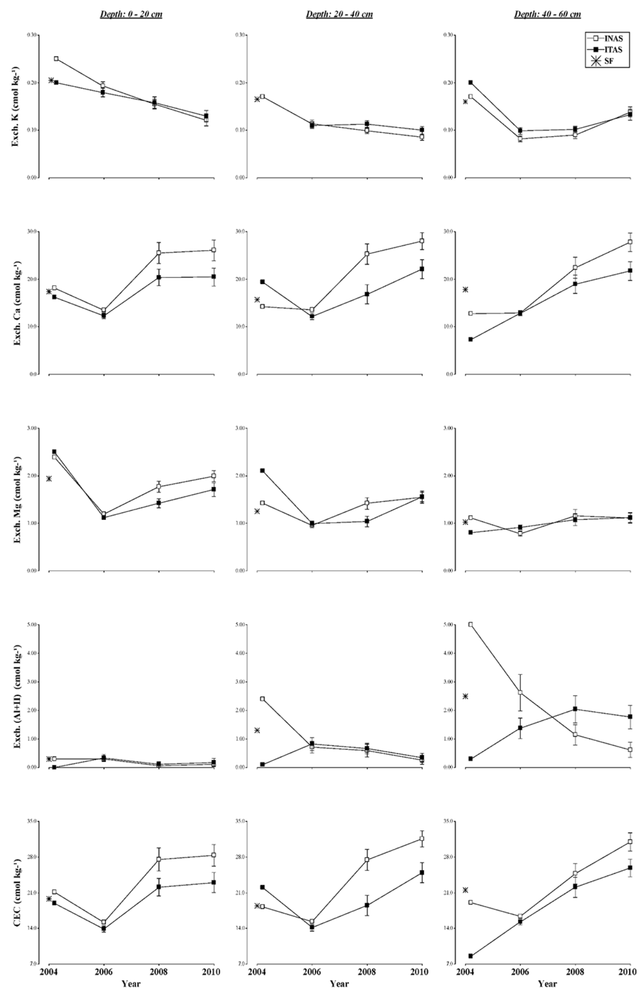

Fig 6. Changes in soil chemical properties. Under long term agroforestry management systems (INAS, ITAS) as compared to soil properties of SF at different depths (0–20, 20–40, 40–60 cm) during 2004 to 2010 experimental period.

doi:10.1371/journal.pone.0132147.g006

types of crops and management practices employed [12, 39]. Reduction in BD is an indication of less compaction and higher porosity; similar changes have been reported by Amusan et al, [40] who found significant differences between bulk density values for different land uses, in cacao plantation (1.32 g cm^{-3}) as compared to SF (1.49 g cm^{-3}).

Porosity. Unlike BD, the porosity was higher in the INAS system and significantly different from ITAS in different years and soil depths assessed (Table 4). The highest porosity values were recorded at 2010 in INAS and in 2008 for ITAS. (Fig 3). The soil physical soil properties can be altered over time by the management practices and nature of vegetative cover. These findings support earlier work by Amusan et al, [40], who reported inverse relationships between BD and porosity of soils under different cropping systems. Cultural methods used in establishment of INAS and ITAS such as digging of good sized pits for root stock planting, and refilling the pits with organic and inorganic fertilizers contributed to improved soil structure. Increased development of roots of cacao and tree crops and frequent litter fall kept the soil in INAS system constantly protected thereby resulting in improved soil physical properties.

Field capacity (FC), wilting point (WP) and plant available water (PAW). The cacao management systems during the years of assessment significantly affected the FC and WP in

Table 4. Soil Physical Properties: BD, Po, FC, WP, and PAW, Influenced by System of Cacao Management and Year of Cultivation (Irrespective of Soil Depth and Genotypes).

Depth	Soil physical properties	Year										ANOVAs of linear mixed effect model with repeated measures		
												Source of variation		
		2004*		2006		2008		2010		Average (2004–2010)		System (S)	Year (Y)	S x Y
		INAS	ITAS	INAS	ITAS	INAS	ITAS	INAS	ITAS	INAS	ITAS		Df	
												1	3	3
													P values	
0–20 cm	BD	1.35 b**	1.42 a	1.32 c	1.36 b	1.30 c	1.33 c	1.26 d	1.35 b	1.31 B***	1.37 A	<0.0001	<0.0001	0.0416
	Po	49.10 c	46.40 d	50.36 b	48.58 c	51.11 b	49.98 b	52.40 a	49.21 c	50.74 A	48.54 B	<0.0001	<0.0001	0.0404
	FC	34.70 d	29.50 e	41.17 b	37.89 c	41.76 b	40.33 b	43.43 a	38.45 c	40.26 A	36.54 B	<0.0001	<0.0001	0.0157
	WP	20.80 d	17.70 e	29.30 b	26.40 c	30.50 b	28.85 b	32.89 a	27.20 c	28.37 A	25.04 B	<0.0001	<0.0001	0.0455
	PAW	13.90 a	11.80 b	11.86 b	11.49 b	11.26 b	11.48 b	10.55 c	11.24 b	11.89 A	11.50 B	0.0025	<0.0001	<0.0001
20–40 cm	BD	1.39 a	1.39 a	1.32 b	1.37 a	1.31 b	1.33 b	1.25 c	1.37 a	1.32 B	1.36 A	<0.0001	<0.0001	0.0001
	Po	47.50 c	47.50 c	50.36 b	48.28 c	50.65 b	49.91 b	52.94 a	48.44 c	50.37 A	48.53 B	<0.0001	<0.0001	0.0001
	FC	39.30 c	33.00 d	43.10 b	40.64 c	42.24 b	42.48 b	45.35 a	40.40 c	42.50 A	39.13 B	<0.0001	<0.0001	<0.0001
	WP	26.50 c	19.60 d	31.14 b	29.06 b	30.43 b	30.90 b	34.03 a	28.95 b	30.53 A	27.13 B	<0.0001	<0.0001	<0.0001
	PAW	12.80 b	13.40 a	11.95 c	11.58 d	11.81 c	11.58 d	11.32 d	11.45 d	11.97 A	12.00 A	0.6919	<0.0001	0.0003
40–60 cm	BD	1.38 b	1.44 a	1.33 c	1.35 c	1.32 c	1.31 c	1.25 d	1.34 c	1.32 B	1.36 A	0.0001	<0.0001	0.0021
	Po	47.90 c	45.70 d	49.78 b	48.92 b	50.05 b	50.62 b	52.80 a	49.44 b	50.13 A	48.67 B	0.0001	<0.0001	0.0022
	FC	41.20 b	30.70 c	42.91 b	41.86 b	42.07 b	44.05 a	45.88 a	42.65 b	43.02 A	39.82 B	<0.0001	<0.0001	<0.0001
	WP	28.70 b	16.90 c	30.66 b	30.23 b	30.14 b	32.38 a	34.16 a	30.92 b	30.92 A	27.61 B	<0.0001	<0.0001	<0.0001
	PAW	12.50 b	13.80 a	12.25 b	11.63 c	11.93 c	11.67 c	11.73 c	11.73 c	12.10 A	12.21 A	0.1764	<0.0001	<0.0001

*, At the time of install of INAS and ITAS. INAS: improved natural agroforestry system, ITAS: improved traditional agroforestry system. BD: bulk density (g cm^{-3}), Po: Porosity (%), FC: field capacity (%), WP: wilting point (%), PAW: plant available water (%).

**Different lower case letters on the right of each value in row indicate significant difference between years 2004 to 2010 and between system per year (0-20cm, 20-40cm, 40–60 depth) (DGC test, P, 0.05).

***Different capital letters on the right of each average value in row for each variable indicate significant difference between systems (DGC test, P, 0.05). *P* value and degree of freedom (*Df*) of fixed effect in linear mixed effect models with repeated measures analysis: *System (S)* (n = 2), *Year (Y)* (n = 4) and *S x Y* (n = 8), random factor in the model: *Block* (n = 3) and *Genotypes* (n = 11). The Depth was analyzed individually.

doi:10.1371/journal.pone.0132147.t004

the top 0–40 cm depth (Table 4). The values of FC and WP were higher in INAS than in ITAS and, overall in both systems, have the tendency to increase with management time (Fig 3). These soil properties were linked to the soil moisture content as well as the clay content of the soil, so that a higher content of organic matter and clay will increase the field capacity. The wilting point refers to the moisture content of soil where the absorptive capacity of the root is less than the demand of the plant [41]. A greater amount of organic matter reduces the wilting point, while the predominance of clay content increases it. The difference between these two soil parameters gives an indication of PAW, which is obtained from the equation PAW = (FC—WP) [38]. In the early years, PAW was higher in the surface layer in INAS than in ITAS. In both systems of cacao management PAW tended to decline with years, even though the change in SOM was minimal (Fig 4).

Effects of cacao genotypes on soil physical properties

Even though the management systems had significant ($P \leq 0.05$) effects on the surface layer (0–20 cm) soil physical properties (BD, Porosity, FC, WP, PAW), but genotypes had no significant effects on soil physical properties (Table 5). Soil physical properties however tended to be influenced by cacao genotypes. Overall in both systems the soils BD were lower than the values at the start of the study. Soil BD values in ITAS were higher than in INAS under all cacao genotypes (Table 5, Fig 3), and the soil BD in ITAS ranged from 1.31 g cm^{-3} (ICS-95) to 1.38 g cm^{-3} (U-30 and Hybrid) whereas BD in INAS ranged from 1.21 g cm^{-3} (CCN-51) to 1.3 g cm^{-3} (ICS-95 and ICT-1026). Such variations may be due to characteristics of the genotypes (root systems, litter fall) and the management practices as is the case of ITAS where soils are compacted and led to higher soil BD [42]. Genotypes with larger root systems are known modify soil physical properties [43].

The best soil porosity was found under genotype CCN-51 in INAS and under genotype ICS-95 in ITAS. This indicates that these genotypes are likely to have greater root systems; thereby these genotypes might have improved soil porosity [43]. Irrespective of genotypes, porosity, FC, and WP were higher in INAS than in ITAS, while PAW was slightly less in INAS than in ITAS (Table 5).

Effects of INAS and ITAS cacao management systems on soil Chemical properties

The chemical properties measured in INAS and ITAS systems of cacao management over a six year period were: pH, electrical conductivity (EC), soil organic matter (SOM), extractable ions (P, K, Fe, Cu, Zn, Mn), exchangeable cations (K, Ca, Mg, Al+H). Based on these values the cation exchange capacity (CEC) was computed. The average values for these properties is showed in S1 Table and values with *P* values calculated from linear mixed effect models with repeated measures for soil chemical properties for the two cacao management systems and over times at three soil depths are presented in Tables 6 and 7. Values for these chemical properties for SF are given in Table 1. Over all changes in chemical properties at varying soil depths of two management systems at different time frames (2004 to 2010) and values for natural forest are shown in Figs 4, 5 and 6.

pH. The soil pH in INAS and ITAS were within the range of a medium to strongly acidic reaction (Fig 4). Significant differences in pH were observed between the systems of management, and between years of assessment (Table 6). In INAS, soil pH values increased slightly by time and were higher in the surface than in the lower soil layers. Overall, pH values in INAS were significantly higher than ITAS. The soil pH at the start of the INAS system was 5.65 (2004) and increased at the end of study (2010) to 5.86 at 0–20 cm, 5.73 at 20–40 cm, and

Table 5. Soil Physical Properties: BD, Po, FC, WP, and PAW, for 2010 Influenced by Systems of Cacao Management and Cacao Genotypes at 0-20cm Depth.

Year	System	Genotypes	Soil chemical properties				
			BD	Por	FC	WP	PAW
**2004***							
	INAS		1.35	49.10	34.70	20.80	13.90
	ITAS		1.42	46.40	29.50	17.70	11.80
**2010**							
	INAS	ICS-95	1.30 a**	51.07 b	42.97 a	33.03 a	9.93 b
		UF-613	1.25 b	52.70 a	43.73 a	33.80 a	9.93 b
		CCN-51	1.21 b	54.20 a	43.97 a	34.43 a	9.53 b
		ICT-1112	1.27 b	52.10 a	42.53 a	31.47 a	11.07 a
		ICT-1026	1.30 a	50.93 b	42.37 a	30.90 a	11.47 a
		ICT-2162	1.27 b	52.07 a	42.73 a	31.63 a	11.10 a
		ICT-2171	1.26 b	52.57 a	44.33 a	33.20 a	11.13 a
		ICT-2142	1.25 b	52.70 a	44.30 a	34.03 a	10.27 b
		H-35	1.23 b	53.70 a	44.03 a	33.90 a	10.13 b
		U-30	1.27 b	52.20 a	43.63 a	33.03 a	10.60 b
		Hybrid	1.27 b	52.20 a	43.17 a	32.33 a	10.83 b
	ITAS	ICS-95	1.31 a	50.43 b	39.97 a	28.47 b	11.50 a
		UF-613	1.32 a	50.07 b	40.83 a	29.27 b	11.57 a
		CCN-51	1.34 a	49.57 b	37.97 b	27.57 b	10.40 b
		ICT-1112	1.36 a	48.53 b	37.97 b	26.07 b	11.90 a
		ICT-1026	1.37 a	48.33 b	38.20 b	26.20 b	12.00 a
		ICT-2162	1.32 a	50.30 b	41.00 a	29.20 b	11.80 a
		ICT-2171	1.35 a	49.20 b	36.93 b	27.00 b	9.93 b
		ICT-2142	1.34 a	49.53 b	37.13 b	26.80 b	10.33 b
		H-35	1.34 a	49.30 b	40.30 a	28.93 b	11.37 a
		U-30	1.38 a	48.07 b	36.03 b	24.47 b	11.57 a
		Hybrid	1.38 a	47.93 b	36.57 b	25.27 b	11.30 a
	Average (INAS)		1.26 B***	52.40 A	43.43 A	32.89 A	10.55 B
	Average (ITAS)		1.35 A	49.21 B	38.45 B	27.20 B	11.24 A
**ANOVAs of linear mixed effect model**							
Source of variability		Df	P value				
System (S)		1	<0.0001	<0.0001	0.0002	<0.0001	0.0074
Genotype (G)		10	0.9371	0.9402	0.9953	0.9725	0.1161
S x G		10	0.9745	0.9749	0.9851	0.9970	0.5351

* At the time of install of INAS and ITAS. INAS: improved natural agroforestry system, ITAS: improved traditional agroforestry system. BD: bulk density (g cm^{-3}), Po: Porosity (%), FC: field capacity (%), WP: wilting point (%), PAW: plant available water (%).

**Different lower case letters on the right of each value in column indicate significant difference between *Genotypes* in 2010 at 0-20cm depth, (DGC test, P, 0.05).

***Different capital letters on the right of each average value for each variable in column indicate significant difference between systems (DGC test, P, 0.05). *P* value and degree of freedom (*Df*) of fixed effect in linear mixed effect models analysis: *System (S)* (n = 2), *Genotype (G)* (n = 11) and *S x G* (n = 22), random factor in the model: *Block* (n = 3).

doi:10.1371/journal.pone.0132147.t005

5.70 at 40–60 cm. In ITAS, the soil pH increased significantly from the initial value of 5.65 to 6.30 after burn in all depths from this point at the end of study (2010) decreased significantly

Table 6. Soil Chemical Properties: pH, EC, SOM, Ext. P, Ext. K, Ext. Fe and Ext. Cu, Influenced by System of Cacao Management and Year of Cultivation (Irrespective of Soil Depth and Genotypes).

Depth	Soil chemical properties	Year /System										ANOVAs of linear mixed effect model with repeated measures		
												Source of variation		
		2004*		2006		2008		2010		Average (2004–2010)		System (S)	Year (Y)	S x Y
		INAF	ITA	IN	IT	IN	IT	IN	IT	INAS	ITA	Df		
												1	3	3
												P values		
0–20 cm	pH	5.60 c**	6.30 a	5.75 b	5.25 d	5.83 b	5.22 d	5.86 b	5.48 c	5.76 A***	5.56 B	0.0007	<0.0001	<0.0001
	EC	0.58 a	0.50 b	0.55 a	0.38 c	0.27 d	0.20 e	0.21 e	0.12 f	0.41 A	0.30 B	<0.0001	<0.0001	0.0667
	SOM	3.90 a	3.50 b	3.52 b	3.28 b	3.20 b	3.47 b	3.34 b	3.38 b	3.49 A	3.41 A	0.3542	0.0088	0.0312
	Ext. P	6.50 c	10.50 a	6.87 c	9.32 b	1.73 e	3.69 d	2.58 e	4.76 d	4.42 B	7.07 A	<0.0001	<0.0001	0.0293
	Ext. K	130.0 a	106.0 b	77.79 c	70.39 d	62.42 d	65.82 d	47.00 e	53.09 e	79.30 A	73.82 B	0.0315	<0.0001	0.0001
	Ext. Fe	69.50 c	182.0 a	50.72 c	91.10 b	55.45 c	114.8 b	37.18 c	51.05 c	53.21 B	109.74 A	<0.0001	<0.0001	<0.0001
	Ext. Cu	1.30 d	2.60 a	2.26 b	1.70 c	1.54 c	2.55 a	2.07 b	1.09 d	1.79 B	1.99 A	0.0074	<0.0001	<0.0001
20–40 cm	pH	5.00 b	5.80 a	5.63 a	5.07 b	5.50 a	5.05 b	5.73 a	5.30 a	5.46 A	5.31 A	0.0570	0.2183	<0.0001
	EC	0.54 a	0.39 b	0.38 b	0.29 c	0.14 d	0.10 e	0.14 d	0.08 e	0.30 A	0.21 B	<0.0001	<0.0001	0.0196
	SOM	1.60 b	3.30 a	1.51 b	1.68 b	1.62 b	1.74 b	1.53 b	1.60 b	1.57 B	2.08 A	<0.0001	<0.0001	<0.0001
	Ext. P	4.10 c	7.00 a	3.11 d	5.34 b	0.65 f	0.99 f	2.09 e	2.58 e	2.49 B	3.98 A	<0.0001	<0.0001	<0.0001
	Ext. K	77.00 b	93.30 a	45.08 c	42.14 c	40.88 c	45.39 c	34.42 d	39.88 c	49.35 B	55.18 A	0.0006	<0.0001	0.0009
	Ext. Fe	150.3 b	329.4 a	50.78 d	92.98 c	58.56 d	114.5 c	22.91 e	71.30 d	70.63 B	152.0 A	<0.0001	<0.0001	<0.0001
	Ext. Cu	1.40 c	1.85 b	2.23 a	1.66 b	1.38 c	2.39 a	1.64 b	1.46 c	1.66 B	1.84 A	0.0092	<0.0001	<0.0001
40–60 cm	pH	4.80 b	5.73 a	5.69 a	5.14 b	5.60 a	5.07 b	5.70 a	5.13 b	5.45 A	5.27 A	0.0509	0.6195	<0.0001
	EC	0.40 a	0.19 d	0.34 b	0.26 c	0.13 e	0.08 e	0.17 d	0.10 e	0.26 A	0.16 B	<0.0001	<0.0001	0.0003
	SOM	1.30 b	2.30 a	0.83 d	1.12 c	1.02 c	1.13 c	0.95 c	1.13 c	1.03 B	1.42 A	<0.0001	<0.0001	<0.0001
	Ext. P	3.30 b	4.90 a	2.56 b	3.81 b	0.66 c	1.12 c	4.56 a	4.38 a	2.77 B	3.55 A	0.0029	<0.0001	0.0702
	Ext. K	76.00 b	103.0 a	35.47 d	38.50 d	37.97 d	46.61 c	53.94 c	54.70 c	50.84 A	60.70 B	<0.0001	<0.0001	0.0003
	Ext. Fe	68.60 a	93.00 a	33.60 b	79.36 a	36.06 b	94.48 a	40.54 b	48.44 b	44.70 B	78.82 A	<0.0001	<0.0001	0.0055
	Ext. Cu	1.30 d	1.30 d	1.83 b	1.57 c	1.14 d	2.28 a	2.02 b	1.36 d	1.57 A	1.63 A	0.4297	<0.0001	<0.0001

*, At the time of install of INAS and ITAS. INAS: improved natural agroforestry system, ITAS: improved traditional agroforestry system pH (1:1), EC: Electric conductivity (dS m^{-1}), SOM: soil organic matter (%). Ext. = Extactable. P (μg g^{-1}), K (μg g^{-1}), Fe (μg g^{-1}), Cu (μg g^{-1}).

**Different lower case letters on the right of each value in row indicate significant difference between years 2004 to 2010 and between system per year (0-20cm, 20-40cm, 40–60 depth) (DGC test, P, 0.05).

***Different capital letters on the right of each average value in row for each variable indicate significant difference between systems (DGC test, P, 0.05). *P* value and degree of freedom (*Df*) of fixed effect in linear mixed effect models with repeated measures analysis: *System (S)* (n = 2), *Year (Y)* (n = 4) and *S x Y* (n = 8), random factor in the model: *Block* (n = 3) and *Genotypes* (n = 11). The Depth was analyzed individually.

doi:10.1371/journal.pone.0132147.t006

Table 7. Soil Chemical Properties: Ext Zn, Ext Mn, Exch K, Exch Ca, Exch Mg, Exch Al+H, and CEC, Influenced by System of Cacao Management and Year of Cultivation (Irrespective of Soil Depth and Genotypes).

Depth	Soil chemical properties	Year /System										ANOVAs of linear mixed effect model with repeated measures		
												Source of variation		
		2004*		2006		2008		2010		Average (2004–2010)		System (S)	Year (Y)	S x Y
		INA	IT	INA	IT	IN	IT	IN	IT	INAS	IT	Df		
												1	3	3
												P values		
0–20 cm	Ext. Zn	2.30 a**	1.73 b	2.40 a	2.06 b	1.63 b	1.92 b	0.94 c	0.67 c	1.82 A***	1.60 B	0.0078	<0.0001	0.0027
	Ext. Mn	17.30 c	5.23 c	73.06 a	73.39 a	37.23 b	28.83 b	20.50 c	11.08 c	37.02 A	29.63 B	0.0173	<0.0001	0.5181
	Exch. K	0.25 a	0.20 b	0.20 b	0.19 b	0.16 c	0.17 c	0.12 d	0.14 d	0.18 A	0.17 A	0.1678	<0.0001	0.0038
	Exch. Ca	18.15 c	16.20 c	13.49 d	12.93 d	23.21 b	11.06 d	25.85 a	20.95 b	20.17 A	15.29 B	<0.0001	<0.0001	<0.0001
	Exch. Mg	2.39 a	2.50 a	1.19 c	1.12 c	1.92 b	1.03 c	2.36 a	1.83 b	1.97 A	1.62 B	<0.0001	<0.0001	<0.0001
	Exch. Al	0.30 b	0.00 b	0.29 b	0.26 b	0.50 a	0.12 b	0.13 b	0.18 b	0.31 A	0.14 B	0.0277	0.3099	0.1489
	CEC	21.09 b	18.90 c	15.17 d	14.50 d	25.79 a	12.38 d	28.46 a	23.09 b	22.63 A	17.22 B	<0.0001	<0.0001	<0.0001
20–40 cm	Ext. Zn	2.00 b	3.10 a	1.29 c	1.08 c	0.73 d	1.09 c	0.55 d	0.37 e	1.14 B	1.41 A	<0.0001	<0.0001	<0.0001
	Ext. Mn	13.80 d	4.35 d	50.11 b	63.92 a	42.51 b	28.06 c	12.74 d	9.54 d	29.79 A	26.46 A	0.262	<0.0001	0.0054
	Exch. K	0.17 b	0.40 a	0.12 c	0.11 c	0.10 c	0.12 c	0.09 d	0.10 c	0.12 B	0.18 A	<0.0001	<0.0001	<0.0001
	Exch. Ca	14.23 c	19.40 b	12.81 c	12.76 c	22.08 b	10.73 c	25.01 a	21.75 b	18.53 A	16.16 B	0.0038	<0.0001	<0.0001
	Exch. Mg	1.42 b	2.10 a	0.96 c	1.00 c	1.47 b	0.87 c	1.60 b	1.60 b	1.36 A	1.39 A	0.5742	<0.0001	<0.0001
	Exch. Al	2.40 a	0.10 c	1.66 b	0.55 c	1.32 b	0.52 c	1.83 b	0.32 c	1.80 A	0.37 B	<0.0001	0.6596	0.0282
	CEC	18.22 c	22.00 b	15.55 d	14.41 d	24.98 b	12.25 d	28.53 a	23.77 b	21.82 A	18.11 B	<0.0001	<0.0001	<0.0001
40–60 cm	Ext. Zn	1.70 a	1.21 b	1.14 b	0.87 c	0.61 d	1.27 b	1.00 c	0.56 d	1.11 A	0.98 B	0.0278	<0.0001	<0.0001
	Ext. Mn	10.90 b	1.83 c	32.81[a]	48.44 a	40.13[a]	19.12 b	18.53 b	12.45 b	25.6 A	20.46 A	0.1155	<0.0001	0.0011
	Exch. K	0.17 b	0.20 a	0.09 d	0.10 d	0.10 d	0.12 c	0.14 c	0.14 c	0.13 B	0.14 A	0.0332	<0.0001	0.4316
	Exch. Ca	12.76 b	7.30 c	12.45 b	12.83 b	20.39 a	11.37 b	24.28 a	21.77 a	17.47 A	13.32 B	<0.0001	<0.0001	0.001
	Exch. Mg	1.11 a	0.80 b	0.78 b	0.92 b	1.22[a]	0.90 b	1.11 a	1.12 a	1.06 A	0.93 B	0.0151	0.0012	0.0017
	Exch. Al	5.00 a	0.30 c	3.25 b	1.37 c	2.83 b	1.47 c	3.19 b	1.57 c	3.57 A	1.18 B	<0.0001	0.7632	0.0015
	CEC	19.04 c	8.60 e	16.58 d	15.22 d	24.54 b	13.85 d	28.72 a	24.60 b	22.22 A	15.57 B	<0.0001	<0.0001	<0.0001

* At the time of install of INAS and ITAS. INAS: improved natural agroforestry system, ITAS: improved traditional agroforestry system, Ext. = Extactable. Zn: (μg g^{-1}), and Mn (μg g^{-1}). Exch. = Exchangeable. K (cmol kg^{-1}), Ca (cmol kg^{-1}), Mg (cmol kg^{-1}) and Al+H (cmol kg^{-1}). CEC: Cationic Exchange Capacity(cmol kg^{-1}).

** Different lower case letters on the right of each value in row indicate significant difference between years 2004 to 2010 and between system per year (0-20cm, 20-40cm, 40–60 depth) (DGC test, P, 0.05).

***Different capital letters on the right of each average value in row for each variable indicate significant difference between systems (DGC test, P, 0.05). *P* value and degree of freedom (*Df*) of fixed effect in linear mixed effect models with repeated measures analysis: *System (S)* (n = 2), *Year (Y)* (n = 4) and *S x Y* (n = 8), random factor in the model: *Block* (n = 3) and *Genotypes* (n = 11). The depth was analyzed individually.

doi:10.1371/journal.pone.0132147.t007

to 5.48 at 0–20 cm, 5.30 at 20–40 cm and 5.13 at 40-60cm (Table 6 and Fig 4). It is generally expected that pH will increase after a burn [44, 45, 46], due to OH-losses, oxide formation, and release of alkaline cations by the ashes [47]. The pH increase measured immediately after burn is in concurrence with data reported by Granged et al. [45, 46], who found that pH increased significantly in soils containing 3.1% organic matter and 41.9% sand before exposure to temperatures of 200–500°C. In the present study the pH values obtained two weeks after burn for ITAS were increased to 6.30, 5.80, and 5.73 (Table 6, Fig 4) for each depth respectively. Our findings of increased soil pH after burn of surface vegetation agreed with the earlier findings of Ekinci [48], who observed that pH changes persisted only for 2 weeks after burn in non calcareous soils.

The differences in soil pH between INAS and ITAS may be related to differences in the dynamics of the soil organic matter [49]. Previously it has been reported that changes in soil pH over time depends on soil properties, vegetative cover, deposition of acidifying materials and weather conditions [50, 51, 52, 53]. Soil pH has a great influence on the solubility of minerals and nutrient availability. It is also a useful indicator of other parameters such as the availability of exchangeable cations (Ca, Mg, K) [54] and micronutrients [55]. Agroforestry systems adapted in the study have greater buffering capacity and that leads to increases in soil pH, and by having perennial vegetative cover with abundant foliage, which provides a permanent soil cover and abundant yearly addition of leaf litter that protects the soil from erosion and minimizes the nutrient loss by surface run-off and leaching. Woody plants with a dense and deep root system are an efficient mechanism for capturing nutrients and offsetting the losses by leaching [56, 57].

Electric Conductivity (EC). Management systems and year of assessments and soil depths had significant effects on soil electrical conductivity (EC) (Table 6). All values of EC were low in both systems of management (<1 dS m^{-1}) as compared to SF (Table 1) and given the high soil moisture content during rainy periods it is expected that such low EC will not be a problem because the EC values even though low but they are at adequate range [26]. In both the systems of management EC decreased considerably with years at all three soil depths from 2004 to 2010. Mean EC in ITAS increased 16.3% and 2.6% after the burn at 0–20 cm and 20–40 cm depths respectively compared to the installation data (Table 1, Table 6), and then decreased by the end of the experiment until EC was 72.1% and 78.9% less for 0-20cm and 20–40 cm depths respectively (Table 6, Fig 4). Similar changes have been observed after natural wildfires by Pardini et al. [58] and by Badía and Martí [59] and in soils after exposure to fire in experimental conditions by Granged et al. [46]. The increase of EC after a burn is a consequence of the soluble inorganic ions that are released during the combustion of soil organic matter, also ashes originated during combustion by organic matter help to release soluble salts that contribute to an increase in pH, EC and CEC [46], Increased EC also occurs after a burn because the exposed soil (without vegetation cover) increases ascending capillary movement from the deeper layers of the profile to the soil surface where the water evaporates and the salts precipitate and gradually accumulate [60]. In both management systems EC decreased at 40-60cm depth each year (Fig 4), possibly because less SOM and ions are retained in the soil solution. On the other hand, in INAS, EC increased 27.9% from 2004 to 2006 and decreased 51.2% at 2010 for 0-20cm depth; at 20–40 cm the EC was nearly the same in 2004 and 2006, and then decreased 63.2% at 2010 (Fig 4). This is probably due to the development of the SF which tends to resemble an ecological or organic agroecosystem [23].

Soil Organic Matter (SOM). The system of management had significant effects on SOM at the deeper soil depths with a few exceptions in the soil surface layer in the early years of assessments (Table 6). In both systems of management, the SOM showed medium levels in the 0–20 cm depth and reduced even further with increasing depths. The values of SOM for both

INAS and ITAS were over 3.0% and less than 4.5% in the surface layer which is optimal for the good development of cacao ([61], Fig 4). In the installation of ITAS the SOM slightly decrease after the burn at 0-20cm depth, while at 20-40cm and 40-60cm depth increased (Table 6), many works support the results at 0-20cm where SOM decreased after combustion in the very short-term [47, 48]. In contrast, others have reported no significant differences in the long-term between burned and unburned soils [62, 63], which supports our results. The increased SOM contents may be due to different causes, such as incorporation of charcoal and hydrophobic organic matter and invasion of N-fixing vegetation [46, 64], increases in black carbon [65], or even via roots [66]. Overall, ITAS recorded higher SOM than INAS. This is probably reflection of higher rates of decomposition/mineralization of vegetative material in the ITAS system where residues of native vegetation were burnt in situ at the start of this system and vegetative cover was more sparse in the early years of establishment that resulted in higher soil temperatures due to greater penetration of solar radiation and higher microbial activities and mineralization of SOM and lower immobilization, combined with other improved chemical, physical and environmental factors, which are important in the stability of organic matter, and availability of nutrients for the plant [67, 68]. At all soil depths, SOM found at the establishment of the experiment was lower than those observed after installation of INAS and ITAS systems. In both systems of management, the surface soil layer recorded the highest amount of SOM because SOM in soil surface includes litter fall, residues, animals and microorganisms at various stages of decomposition [54]. The organic matter positively influences nearly all important properties that contribute to the quality of soil [69]. Thus, it is crucial to understand and emphasize the key importance of crops and soil management to maintain and increase the organic matter content of the soil in order to develop good soil quality [70].

Extractable P. Extractable P content was significantly affected by system of management and years of assessment at all soil depths (Table 6). In ITAS, the Ext. P after burn increased in all depths (Fig 4), then decreased significantly until 2008 and then the tendency was to recover the initial values. In the first 20 cm of soil depth, both systems of management started in 2004 with similar Ext. P (6.5 μg g^{-1}). After burn, Ext. P increased to 10.5 μg g^{-1} in ITAS, then declined until 2008 for both systems (1.73 μg g^{-1} and 3.69 μg g^{-1} for INAS and ITAS, respectively) and recovers in 2010 (2.58 μg g^{-1} and 4.76 μg g^{-1} for INAS and ITAS, respectively) (Table 6). The significant increase of Ext. P in ITAS after the burn may be a result of the strong mineralizing effect of fire on organic P [71]. Higher values of Ext. P found in ITAS could be the result of slash and burn in situ of SF trees. Ash deposits after burning of biomass help to fertilize the soil by rapidly releasing mineral nutrients such as Ext. Ca, Mg, P and K for crop use [68, 72, 73, 74, 75]. These favorable changes in the extractable soil nutrients due to conversion of forest system through slash and burn methods is known to persist for at least six years [74]. In addition, fluctuations in soil Ext. P may be caused by characteristics of the soil where there is greater P fixation or organic compounds formed. The decomposition process can increase the availability of P in acid soils by blocking the P adsorption sites on soil mineral matter and aluminum complexes. In ITAS and INAS the available P decreased with increasing soil depth (Fig 4). These results confirm the observation made by He et al. [42] and Wang et al. [76] that in no-till systems P levels at the 0–10 cm depth are greater than in the deeper layers; on the other hand, those results were in contrast with what was found by Amusan et al. [40], who reported the best values in a system of cacao versus SF.

Extractable K. The levels of Ext. K were low in both management systems at all soil depths and years of assessment (Table 6, Fig 4). Ext. K in all the soil depths and over time frame was significantly higher in 2004 in both management systems than at the end of the experiment. In both management systems, the year of management had a significant effect on soil Ext. K (Table 6). In ITAS, Ext. K increased among 20 to 60cm depth due to burning of vegetative

matter (Fig 4), similar to the Ext. P and for the same reasons [68, 72, 73, 74, 75]. The Ext. K then decreased considerably by 2006 and the values were maintained with low variation until the end of the experiment. The level of Ext. K in both systems of management and at all soil depths declined with the increase in years of assessment. Such a decline in soil Ext. K could be explained to the fact that the amount of K absorbed by plants is higher than the K present and replenished in the soil by slow transfer of K from primary minerals to soil exchange complexes, the soil solution and K released from mineralization of SOM [67]. In addition, Ext. K is a fairly mobile ion which is easily lost through runoff and leaching from soil. Potassium is also retained in litter accumulated on the soil surface and is released slowly as organic matter decomposes [67]. A high content of polyphenols and lignins also slows the decomposition process [77].

Extractable micronutrients Fe, Cu, Zn and Mn. The cacao management system had a significant influence on extractable Fe in all depths, on extractable Cu at 20–40 cm depth (Table 6) and extractable Zn until 40cm depth (Table 7). The years of assessment influenced significantly the extractable Fe, Cu (Table 6), Zn and Mn (Table 7) in all depths. Overall, ITAS recorded increased values for extractable Fe and Cu at all depths after burn (Fig 5). Many studies report the increase of micronutrients after a burn [71, 78, 79], Differences are probably due to variations in fire intensity, precipitation regime, vegetation type and soil type. Two years after the burn the available Fe declined considerably in all depths coincident with the result usually reported in burned soils [71]. The decrease of extractable Fe might be associated with Fe losses from the eroding sediments [71]. In INAS the levels of extractable Fe and Zn have the overall tendency to decrease with time. Extractable Mn increased after two years then returned to the starting level. The extractable Cu had a higher level of variability; this variation may be possibly influenced by the soil pH [80]. The extractable Mn in all depths showed a significant increase after the installation registering values similar in ITAS and INAS at 0–20 cm in 2006, while the values at 20–40 cm and 40-60cm in ITAS were higher than INAS (Table 7), this dynamic of Mn is supported by Gómez-Rey et al. [71], who found similar results after burning.

Exchangeable Ca, Mg, K. Soil exchangeable Ca was significantly affected by the systems of cacao management at all depths (Table 7). Exchangeable Mg only was influenced by the systems of management at 0-20cm depth and 40-60cm depth, while the exchangeable K and exchangeable (Al+H) was influenced by the systems of management at all depths. The soil exchangeable K, Ca and Mg were affected significantly by the years of experiment (Table 7). In ITAS after the burn exchangeable Mg increased significantly while Ca and K decreased slightly but not significantly, however, exchangeable (Al+H) decreased significantly (Fig 6). An increase of the concentration of available basic cations after the burn was reported by Alegre et al. [81] in the same region as the experiment. Another reason may be due to the accumulation of ashes rich in oxides and carbonates of basic ions [47, 71, 82]. In both systems, the highest concentrations of exchangeable K and Mg were observed in the surface soil layers (Table 7). This is a reflection of the high SOM presence in surface soil layer. Mineralization and immobilization of soil SOM in the surface soil has high amounts of extractable and exchangeable of N, P, K and micronutrients. Soil Organic Matter combined with other soil chemical, physical and environmental factors, which are important in the stability SOM, provides higher availability of nutrients to plant [67].

Exchangeable Al+H

Soils under the SF system were medium to strongly acidic in reaction (Table 1). In this regard, at all the years of assessment in both systems, there were attributably higher concentrations of exchangeable Al+H in the soil at the pH range between 5.25 and 5.86. At deeper soil depths, the systems of management significantly affected the exchangeable Al+H with ITAS being

higher than INAS and the concentrations increased with increasing soil depths (Table 7). The soil under management conditions in accordance with the balance of the ecosystem has better features than one under conventional management [83]. A study carried out by Theodoro et al. [83] found that soils with high organic matter had higher pH and higher availability of Ca, Mg, K, P and Zn and a drop in exchangeable aluminum.

Cationic exchange capacity (CEC). Cationic exchange capacity (CEC) is a measure of the capacity of the soil to adsorb and release cations [55]. It is also a good indicator of mineral soil fertility, which depends on the texture of soil and on the amount of SOM [84]. Significant differences in soil CEC were attributed to the management systems, years of management at all soil depths (Table 7). CEC values were significantly higher in INAS than in ITAS (Fig 6). This situation agrees with that reported by Sharma et al. [21] who found higher values in an agroforestry system compared to an agri-horticultural system, pastoral system and arable land. In both systems of management at all the soil depths, CEC increased with increasing years. Such increases in CEC could be attributed to the greater accumulation of organic matter in cacao production systems with time that promoted greater biodegradation and mineralization of surface biomass. Generally, the greatest accumulation of organic matter in soil leads to changes in the dynamics of macro and microelements, decreases soil acidity, maintains the predominance of cationic exchange capacity and improves microbial activity [74].

Effects of cacao genotypes on soil chemical properties

Mean values for soil chemical properties at 0–20 cm depths under various cacao genotypes in two cacao management systems are presented in Tables 8 and 9. In both the systems of management, cacao genotypes had limited effects on soil chemical properties). However in each system of management cacao genotypes did induce slight changes in soil chemical properties. The *System* x *Genotype* interaction had no significant effects on the measured chemical properties. With exception of SOM, Ext. K and Ext. Fe, the other soil chemical properties have significant differences among *Systems* but any variable measured not have significance among genotypes.

pH. In INAS, the overall surface soil pH among the evaluated cacao genotypes increased slightly in INAS 4.6% in average from the initial soil pH in 2004 (Table 8), with the exception of genotypes CCN-51 and ICT-2162 where the soil pH decreased 5.4% and 0.5% respectively. The values of soil pH under the cacao genotypes in INAS were in the range of 5.30 to 6.22. On the other hand, in ITAS the soil pH under the cacao genotypes decreased on average 13.0% from the initial soil pH determined in 2004. Management has great effect on soil pH than genotypes (Table 6 and Fig 4). Eventhough burn of vegetation is known to increase soil pH but burn effect persist for only for two weeks [48] and difference in soil pH among the two systems of management may be related to nature of organic matter. The increase in soil pH values under cacao trees under INAS are coincident with the report of Barreto et al. [85], where they found high values of soil pH under a cacao crop than a forestry system.

EC. The measured soil EC under cacao genotypes followed similar trend as that of soil pH. Overall soil EC under the cacao genotypes were higher in INAS in comparison with ITAS and EC under both the systems were lower than soil EC (Tables 1 and 8)

SOM. In INAS, ICT-2162 and the hybrid recorded the highest SOM; whereas in ITAS, UF-613 and ICT-2171 recorded the highest SOM (Table 8). Tree species are also known to show differences in SOM [86]. Another reason that the SOM in ITAS was higher than INAS is because the cacao trees apparently have more aerial biomass. There was more competition between cacao and native trees in INAS compared to the cacao trees that grew in ITAS without

Table 8. Soil Chemical Properties: pH, EC, SOM, Ext. P, Ext. K, Ext. Fe and Ext. Cu, for 2010 Influenced by Systems of Cacao Management and Cacao Genotypes at 0-20cm Depth.

Year	System	Genotypes	Soil chemical properties						
			pH	EC	SOM	Ext. P	Ext. K	Ext. Fe	Ext. Cu
**2004***									
	INAS		5.60	0.58	3.90	6.50	130.00	69.50	1.30
	ITAS		6.30	0.50	3.50	10.50	106.00	182.00	2.60
**2010**									
	INAS	ICS-95	5.82 b**	0.16 b	3.00 a	2.57 b	43.67 b	33.03 b	2.27 a
		UF-613	6.11 a	0.24 a	3.51 a	2.31 b	59.67 b	23.13 b	1.83 a
		CCN-51	5.30 b	0.11 b	2.28 b	2.63 b	47.67 b	55.37 b	2.30 a
		ICT-1112	5.87 b	0.24 a	3.00 a	2.79 b	59.00 b	9.73 b	1.83 a
		ICT-1026	6.22 a	0.23 a	3.48 a	4.10 a	32.67 b	5.10 b	1.50 b
		ICT-2162	5.57 b	0.17 b	3.69 a	3.31 b	31.00 b	54.27 b	2.53 a
		ICT-2171	6.22 a	0.26 a	3.63 a	2.72 b	70.67 a	54.83 b	2.43 a
		ICT-2142	6.22 a	0.28 a	3.63 a	2.36 b	53.67 b	53.07 b	2.17 a
		H-35	5.64 b	0.17 b	2.95 a	1.18 b	42.33 b	31.30 b	1.90 a
		U-30	5.71 b	0.30 a	3.59 a	1.98 b	33.67 b	69.43 b	2.13 a
		Hybrid	5.76 b	0.20 b	3.94 a	2.44 b	43.00 b	19.67 b	1.90 a
	ITAS	ICS-95	5.33 b	0.15 b	3.59 a	2.36 b	60.33 b	52.03 b	0.93 b
		UF-613	5.36 b	0.11 b	3.74 a	2.66 b	54.00 b	39.87 b	0.90 b
		CCN-51	5.56 b	0.10 b	3.03 a	6.92 a	43.33 b	46.60 b	0.97 b
		ICT-1112	5.40 b	0.11 b	3.48 a	7.20 a	46.33 b	66.07 b	1.23 b
		ICT-1026	5.48 b	0.10 b	3.45 a	5.05 a	46.00 b	34.13 b	1.00 b
		ICT-2162	5.71 b	0.13 b	3.49 a	4.18 a	55.67 b	56.00 b	1.07 b
		ICT-2171	5.66 b	0.12 b	3.65 a	5.11 a	55.00 b	27.10 b	1.00 b
		ICT-2142	5.61 b	0.19 b	3.44 a	6.37 a	54.67 b	67.10 b	1.47 b
		H-35	5.31 b	0.09 b	3.25 a	4.47 a	46.33 b	40.03 b	1.07 b
		U-30	5.60 b	0.16 b	3.16 a	2.54 b	54.33 b	35.87 b	1.00 b
		Hybrid	5.24 b	0.09 b	2.93 a	5.53 a	68.00a	96.73 a	1.37 b
	Average (INAS)		5.86 A***	0.21 A	3.34 A	2.58 B	47.00A	37.18 B	2.07 A
	Average (INAS)		5.48 B	0.12 B	3.38 A	4.76 A	53.09	51.05 A	1.09B
**ANOVAs of linear mixed effect model**									
Source of variability		Df				P value			
System (S)		1	0.0175	0.0001	0.8237	0.0051	0.3991	0.1235	<0.0001
Genotype (G)		10	0.8994	0.2914	0.7778	0.7472	0.9539	0.6869	0.7524
S x G		10	0.9172	0.8653	0.894	0.8825	0.9448	0.2881	0.6472

* At the time of install of INAS and ITAS. INAS: improved natural agroforestry system, ITAS: improved traditional agroforestry system, pH (1:1), EC: Electric conductivity (dS m^{-1}), SOM: soil organic matter (%), Ext. = Extactable. P (µg g^{-1}), K (µg g^{-1}), Fe (µg g^{-1}), Cu (µg g^{-1}).

**Different lower case letters on the right of each value in column indicate significant difference between *Genotypes* in 2010 at 0-20cm depth, (DGC test, P, 0.05).

*** Different capital letters on the right of each average value for each variable in column indicate significant difference between systems (DGC test, P, 0.05). *P* value and degree of freedom (*Df*) of fixed effect in linear mixed effect models analysis: *System (S)* (n = 2), *Genotype (G)* (n = 11) and *S x G* (n = 22), random factor in the model: *Block* (n = 3).

doi:10.1371/journal.pone.0132147.t008

any competition with trees during the first years of the plantation, where all trees of the native forest were eliminated with the slash and burn method.

Extractable P. Soils under cacao genotypes of ITAS recorded twice the levels of extractable P than the soils under genotypes of the INAS system and extractable P in both the systems were lower than observed for initial extractable P (Table 8). The highest extractable P was found in the soil of genotypes ICT-1026 in INAS (4.1 μg g^{-1}), and genotypes CCN-51 and ICT 1112 in ITAS (6.92 and 7.20 μg g^{-1} respectively). Phillips and Fahey [87] indicate that roots induce higher extractable P, presumably the mycorrhizal fungi and other microbes associated with roots release phosphatase enzymes which help to release organically bound P into plant available P [87]. Positive effects phosphatase enzymes in increasing P mobility in rhizosphere are well documented [87, 88]. In both management systems during the initial years of establishment, a high amount of extractable P was observed (Fig 4), however with increasing time the extractable P in the surface soil layers declined. In the early years of tree establishment, phosphatase enzymes probably played a major role in mobilization of a large part of the organically bound P in SOM [89]. With time SOM was reduced and tree uptake of P was increased thereby reducing the amount of soil extractable P.

Extractable K. Over the years, the amount of extractable K declined under cacao genotypes (Table 8). However the amount of soil extractable K under cacao genotypes varied. The highest extractable K was found in the soil of genotypes ICT-2171 in INAS (70.67 μg g^{-1}) and hybrid in ITAS (68.00 μg g^{-1}). These results are concordant with the results obtained by Alfaia et al. [90] who found lower extractable K in an agroforestry system than in pasture or primary upland forest.

Extractable Fe, Cu, Zn and Mn. In both management systems, the soil extractable Fe and Zn under genotypes were the same statistically, however genotypes showed differential effects on the amount of extractable soil Fe and Zn. In all the soils under genotypes in both systems, we found less extractable Fe and Zn than the initial amounts in 2004 (Tables 8 and 9). The highest amount of extractable Fe was found in the soil of genotypes U-30 in INAS (69.43 μg g^{-1}) and the hybrid in ITAS (96.73 μg g^{-1}). The highest amount of extractable Cu was found in the soil of genotypes ICT-2162 in INAS (2.53 μg g^{-1}) and the ICT-2142 in ITAS (1.47 μg g^{-1}), the values of extractable Cu was significative mayor in INAS than the ITAS. For extractable Zn (Table 9) the highest value was found in soils under genotype ICT-2162 (1.93 μg g^{-1}) in INAS and ICT-2142 (1.10 μg g^{-1}) in ITAS. The highest values of extractable Mn (Table 9) was found in the soils around of genotype ICT-2162 in INAS (43.83 μg g^{-1}) and the hybrid in ITAS (18.03 μg g^{-1}). The amounts of soil extractable Cu and Mn under genotypes in INAS were twice as much as observed under ITAS. This shows that in INAS management system there was higher mobilization of these two micronutrients. In both systems soil extractable Mn varied among genotypes.

Exchangeable cations. In both systems, the soil exchangeable K, Ca and Mg under cacao genotypes were similar statistically (Table 9). The increase of exchangeable Ca in both systems and decrease in some exchangeable Mg in ITAS under various genotypes is concordant with the results obtained by Alfaia et al. [90] who compared soil fertility in three land uses: agroforestry system, pasture and primary upland forest. With respect to soil exchangeable K, under cacao genotypes in both systems of management the decreased value from the initial is concordant with Barreto et al. [91], who found lower values of exchangeable K in cacao crops under "Cabruca" system (similar to INAS) than the Atlantic bush (similar to the SF).

Exchangeable (Al+H). In both systems, soil under cacao genotypes, the exchangeable (Al+H) were similar statistically (Table 9). In both systems, soil under various cacao genotypes the average of exchangeable (Al+H) decreased with time as compared to the native forest (Table 1). The reduction of exchangeable (Al+H) under cacao in INAS was reduced by 56.66% and ITAS by 40.0%. The highest value of exchangeable (Al+H) in INAS was evident in the soil under genotype CCN-51 (0.43 cmol kg^{-1}) and in ITAS was in ICT-1112 (1.47 cmol kg^{-1}). Such

Table 9. Soil Chemical Properties: Ext Zn, Ext Mn, Exch K, Exch Ca, Exch Mg, Exch Al+H, and CEC, for 2010 Influenced by Systems of Cacao Management and Cacao Genotypes at 0-20cm Depth.

Year	System	Genotypes	Soil chemical properties						
			Ext. Zn	Ext. Mn	Exch. K	Exch. Ca	Exch. Mg	Exch. Al+H	CEC
2004*									
	INAS		2.30	17.30	0.25	18.15	2.39	0.30	21.09
	ITAS		1.73	5.23	0.20	16.20	2.50	0.00	18.90
2010									
	INAS	ICS-95	1.03 b**	15.60 b	0.11 b	18.37 b	1.72 b	0.37 b	20.57 b
		UF-613	0.78 b	19.87 b	0.18 a	26.67 b	2.37 b	0.00 b	29.22 b
		CCN-51	0.70 b	27.73 b	0.12 b	17.55 b	2.07 b	0.43 b	20.17 b
		ICT-1112	0.63 b	6.57 b	0.15 b	25.06 b	2.27 b	0.27 b	27.75 b
		ICT-1026	0.67 b	9.00 b	0.08 b	24.15 b	2.10 b	0.00 b	26.34 b
		ICT-2162	1.93 a	43.83 a	0.08 b	22.19 b	3.16 a	0.00 b	25.42 b
		ICT-2171	1.40 b	24.23 b	0.18 a	32.30 a	2.18 b	0.00 b	34.66 a
		ICT-2142	1.27 b	14.83 b	0.14 b	34.04 a	2.44 b	0.00 b	36.62 a
		H-35	0.43 b	18.47 b	0.11 b	25.06 b	1.94 b	0.40 b	27.52 b
		U-30	1.07 b	31.23 b	0.09 b	32.61 a	2.94 a	0.00 b	35.64 a
		Hybrid	0.47 b	14.17 b	0.11 b	26.35 b	2.74 a	0.00 b	29.20 b
	ITAS	ICS-95	0.63 b	6.50 b	0.16 b	20.93 b	2.11 b	0.27 b	23.47 b
		UF-613	0.53 b	10.03 b	0.14 b	22.98 b	2.02 b	0.00 b	25.14 b
		CCN-51	0.40 b	15.17 b	0.11 b	15.12 b	1.54 b	0.00 b	16.77 b
		ICT-1112	0.70 b	11.87 b	0.12 b	20.35 b	1.59 b	1.47 a	23.53 b
		ICT-1026	0.53 b	8.63 b	0.12 b	26.47 b	1.67 b	0.00 b	28.26 b
		ICT-2162	0.47 b	11.40 b	0.14 b	22.94 b	1.78 b	0.00 b	24.86 b
		ICT-2171	0.60 b	10.20 b	0.14 b	25.46 b	2.12 b	0.00 b	27.71 b
		ICT-2142	1.10 b	10.53 b	0.14 b	23.21 b	1.82 b	0.13 b	25.30 b
		H-35	0.57 b	9.87 b	0.12 b	18.09 b	1.75 b	0.00 b	19.96 b
		U-30	0.93 b	9.70 b	0.14 b	20.49 b	1.99 b	0.00 b	22.62 b
		Hybrid	0.90 b	18.03 b	0.17 a	14.39 b	1.77 b	0.07 b	16.40 b
	Average (INAS)		0.94 A***	20.50 A	0.12 B	25.85 A	2.36 A	0.13 B	28.46 A
	Average (ITAS)		0.67B	11.08 B	0.14 A	20.95 B	1.83 B	0.18 A	23.09 B
**ANOVAs of linear mixed effect model**									
Source of variability		Df	P value						
System (S)		1	0.0976	0.0021	0.4795	0.0382	0.0158	0.7816	0.0259
Genotype (G)		10	0.5619	0.2055	0.9229	0.4248	0.8978	0.4050	0.4384
S x G		10	0.5548	0.2476	0.8983	0.8752	0.8889	0.7119	0.8679

* At the time of install of INAS and ITAS. INAS: improved natural agroforestry system, ITAS: improved traditional agroforestry system,Ext. = Extactable. Zn (μg g^{-1}), Mn (μg g^{-1}). Exch. = Exchangeable. K (cmol kg^{-1}), Ca (cmol kg^{-1}), and Mg (cmol kg^{-1}) and Al+H (cmol kg^{-1}). CEC: Cationic Exchange Capacity (cmol kg^{-1}).

**Different lower case letters on the right of each value in column indicate significant difference between Genotypes in 2010 at 0-20cm depth, (DGC test, P, 0.05)

*** Different capital letters on the right of each average value among for each variable in column indicate significant difference between systems (DGC test, P, 0.05). P value and degree of freedom (Df) of fixed effect in linear mixed effect models analysis: System (S) (n = 2), Genotype (G) (n = 11) and S x G (n = 22), random factor in the model: Block (n = 3).

doi:10.1371/journal.pone.0132147.t009

variation in exchange (Al+H) was contrary to the variation of pH. The increasing of exchange (Al+H) is attributed to release of acid exudates in the rhizosphere by tree roots [83].

CEC. In both systems, cacao genotypes did not have significant effects on CEC, but overall soils under genotypes in INAS recorded higher soil CEC than soils under genotypes of ITAS (Table 9). The better CEC in INAS was registered in the soil under genotype ICT-2142 (36.62 cmol kg^{-1}) and U-30 (35.64 cmol kg^{-1}), while in ITAS was better in ICT-1026 (28.26 cmol kg^{-1}); these results are coincident with Barreto et al. [85], who found higher values of CEC in the cacao crops under "Cabruca" system than the Atlantic bush.

Overall, genotypes had some effects on soil chemical properties. Variations in soil chemical properties such as pH, organic-C and rate of N mineralization under different tree species have been reported [91, 92]. Inter-specific differences in soil properties among cacao genotypes might have been caused by exudation of organic acids, quality or quantity of litter deposited and the rate of litter decomposition, and nutrients uptake or pumping of nutrients from deeper soil to surface soil layers [92, 93]. The composition of tree species and environmental condition inside of the tree systems also contributes to the differences in the soil properties [94]

Conclusions

The long term improved natural agroforestry system (INAS) and improved traditional agroforestry systems (ITAS) of cacao genotypes management systems adapted have significant effects on soil physical and chemical properties in Amazon region of Peru. Soil chemical and physical properties in both systems of management appear to be approaching equilibrium with after 6 years of management. The dynamics of soil physical indicators have changed significantly in both systems of management as compared to secondary natural forest (SF) and initial estimates. Years of assessment and depth of soil have significant effects on physical indicators such as bulk density, porosity, field capacity and wilting point, however cacao genotypes overall had minimal effects on the soil physical properties. In both systems of management the SOM content at various soil depths increased with years, however increase of SOM, extractable P, K, and Mg, and exchangeable. K and. Cu was very substantial at the surface soil layer (0-20cm). Overall INAS, had major effect in improving soil pH, CEC, Exch Mg and (Al+H) than ITAS. Longer time is needed to fully understand the impact of natural or traditional agroforestry systems of cacao management on soil properties. The agroforestry system of perennial crop management can play an important role in improving soil fertility by storing large amount of organic carbon in the soil thereby retaining substantial amount of nutrients. Future Research is needed to explore the impact of long term agroforestry management of perennial crops on soil erosion, biodiversity of trees, soil microbes, birds, animals and socio-economic services.

Supporting Information

S1 Table. Average of Data base of physical and chemicals properties of soil under agroforestry system s of cacao in Peruvian Amazon
(XLS)

Acknowledgments

Thanks go to the United States-Department of Agriculture—Agricultural Research Service (USDA-ARS) and US Embassy (INL) in Lima-Peru for their continued support to research and to the extension program in Peru. To Instituto de Cultivos Tropicales [Tropical Crops Institute]—ICT, for providing technical and logistical facilities for the field research and laboratory analytical facilities. To Luis Zuñiga Cernades of ICT and Fernando Volker Puertas Ramos for the information provided and help in the development of this work. We thank Drs

S. C. Rao and Marshall Elson for excellent review of this paper. Finally, thanks to all of our colleagues who supported the undertaking of this work.

Author Contributions

Conceived and designed the experiments: EAG VB. Performed the experiments: EAG MC. Analyzed the data: EAG JA OL VB. Contributed reagents/materials/analysis tools: EAG VB MC AJ. Wrote the paper: EAG VB JA. Design of maps: EAG.

References

1. Willer H, Yussefi M. The world of organic agriculture: statistics and emerging trends 2004. International Federation of Organic Agriculture Movements, Bonn, Germany, 2004. pp. 96–97.
2. OEEE-MINAG. Series históricas de producción agrícola—Compendio estadístico. Available: http://frenteweb.minag.gob.pe/sisca/?mod = consulta_cult. 2014 Accessed 18 September 2014.
3. Alegre JC, Cassel DK. Effects of land-clearing method and soil management on crop production in the Amazon. Field Crops Research 1990, 24: 131–141.
4. Watson RT, Noble IR, Bolin B, Ravindranath NH, Verardo DJ, Dokken DJ. Land use, land-use change and forestry: A Special Report of the IPCC. Cambridge University Press, Cambridge, UK. 2000
5. Flores PS. Agroforestería amazónica: una alternativa a la agricultura migratoria. Capítulo 10. En: Kalliola R, Flores Paitan S, editors. Geoecología y Desarrollo Amazónico. Estudio integrado en la zona de Iquitos, Perú, Turun Yliopisto, Turku, Finland. 1998
6. Olanrewaju OD. Changes in soil properties following conversion of humid tropical rainforest of Nigeria into cocoa agroforests. J. of Applied Biological Sciences. 2008; 2(3): 9–13.
7. Tangjang S, Arunachalam K, Arunachalam A, Shukla AK. Microbial population dynamics of soil under traditional agroforestry systems in northeast India. Research Journal of Soil Biology. 2009, 1: 1–7.
8. Alegre JC, Cassel DK, Bandy DE. Effects of land clearing and subsequent management on soil physical properties. Soil Sci. Soc. Amer. J. 1986; 50: 1379–1384.
9. Alegre J, Arévalo L, Ricse A, Callo-Concha D, Palm C. Secuestramiento de carbono con sistemas alternativos en el Peru In. Sistemas Agroflorestais, Tendência da Agricultura Ecológica nos Tropicos, sustento da vida e sustento de vida.: 27–32 Editores: Muller M., Gama-Rodrigues A., Fontes I. Silva & Carvalho M.. Sociedade Brasileira de sistemas Agroflorestais, Comissão Executiva do plano da lavoura cacaueira y Universidade Estadual do Norte Fluminense. Ilehues- BA. 2006
10. Chepstow-Lusty A, Jonsson P. Inca agroforestry: lessons from the past. Ambio. 2000; 29: 322–328.
11. Rice RA. Agricultural intensification within agroforestry: The case of coffee and wood products. Agriculture, Ecosystem and Environment. 2008; 128: 212–218
12. Lal R. Soil quality and agricultural sustainability. Advances in Soil Science. CRC Press, Boca Raton, Florida. 1998. pp. 17–30
13. Clark MS, Horwath WR, Shennan C, Scow KM. Changes in soil chemical properties resulting from organic and low-input farming practices. Agronomy J. 1998; 90: 662–671.
14. Jose S. Agroforestry for ecosystem services and environmental benefits: an overview. Agroforest Syst. 2009; 76: 1–10
15. Arévalo-Gardini E, Zuñiga CL, Arévalo AC, Adriazola del AJ. Cacao: Manejo integrado del cultivo y transferencia de tecnología. Instituto de Cultivos Tropicales-ICT, Tarapoto, Perú. 2004. 184p.
16. Nair PKR, Nair VD, Kumar BM, Haile SG. Soil carbon sequestration in tropical agroforestry systems: a feasibility appraisal. Environmental Sci & Policy. 2009; 12: 1099–1111.
17. Fassbender HW. Long term studies of soil fertility in cacao-shade trees-agroforestry systems: Results of 15 years of organic matter and nutrients research in Costa Rica. In. Schulte A, Ruhiyat D, editors. Soils of Tropical Forest Ecosystems, Characteristics, Ecology and Management. Springer-Verlag, Berlin. 1998. 207pp.
18. Schroth G, Bede LC, Paiva AO, Cassano CR, Amorim AM, Faria D, et al. Contribution of agroforests to landscape carbon storage. Mitigation and Adaptation Strategies for Global Change. Published on line November 22, 2013.doi: 10.1007/s11027-013-9530-7
19. Somarriba E, Cerda R, Orozco L, Cifuentes M, Dávila H, Espin T, et al. Carbon stocks and cocoa yields in agroforestry systems of Central America. Agric Ecosys Environ. 2013; 173:46–57. doi: 10.1016/j.agee.2013.04.013

20. Jacobi J, Andres C, Schneider M, Pillco M, Calizaya P, Rist S. Carbon stocks, tree diversity, and the role of organic certification in different cocoa production systems in Alto Beni, Bolivia. Agrofor Syst. 2013. doi: 10.1007/ s10457-013-9643-8

21. Sharma KL, Ramachandra-Raju K, Das SK, Prasad-Rao BRC, Kulkarni BS, Srinivas K, et al. Soil fertility and quality assessment under tree-, crop-, and pasture-based land-use systems in a rainfed environment. Comm in Soil Sci and Plant Anal. 2009; 40: 1436–1461.

22. Reganold JP. Comparison of soil properties as influenced by organic and conventional farming systems. American J of Alternative Agric. 1988; 3: 144–155.

23. Drinkwater LE, Letourneau DK, Workneh F, van Bruggen AHC, Shennan C. Fundamental differences between conventional and organic tomato agroecosystems in California. Ecological Applications. 1995; 5: 1098–1112.

24. Werner S. The impact of management practices on species richness within productive rubber agroforests of Indonesia. In: Sist P, Sabogal C, Byron Y. Management of secondary and logged-over forests in Indonesia. (Selected proceedings of an international workshop 17–19 Nov 1997), Center for International Forestry Research, Bongor, Indonesia. 1999. pp. 33–44

25. Holdridge LR, Grenke W, Hatheway WH, Liang T, Tosi JA. Forest environments in tropical life zones: a pilot study. Pergamon Press, Oxford.1971

26. Soil Survey Staff. Keys to soil taxonomy, sixth ed. US Govt. Print. Office, Washington, D.C. 1994. 306 pp.

27. Anderson JM, Ingram JSI. Tropical soil biology and fertility: a handbook of methods. C.A.B. International, Wallingford, UK. 1993. 221 p

28. Saxton KE, Rawls WJ. Soil water characteristic estimates by texture and organic matter for hydrologic solutions. Soil Sci. Soc. Am. J. 2006; 70: 1569–1578.

29. Di Rienzo JA, Casanoves F, Balzarini MG, Gonzalez L, Tablada M, Robledo CW. InfoStat versión 2013. Grupo InfoStat, FCA, Universidad Nacional de Córdoba, Argentina. 2013. Available: http://www.infostat.com.ar. Accessed 18 September 2014.

30. Pinheiro J.C., Bates D.M. Mixed-Effects Models in S and S-PLUS. Springer, New York. 2004

31. Julio A. Di Rienzo JA, Macchiavelli R, Casanoves F. Linear Mixed Models: InfoStat applications. 1rt ed.— Córdoba, Grupo Infostat. 2012. 193p,

32. Gómez S, Torres V, García Y, Navarro JA. Procedimientos estadísticos más utilizados en el análisis de medidas repetidas en el tiempo en el sector agropecuario. Revista Cubana de Ciencia Agrícola. 2012; 46: 1–7

33. Venables WN, Smith DM, and the R Core Team. An Introduction to R. Notes on R: A Programming Environment for Data Analysis and Graphics Version 3.1.1 (2014-07-10). 2014. 105p

34. Igwe CA. Soil physical properties under different management systems and organic matter effects on soil moisture along soil catena in southeastern Nigeria. Tropical and Subtropical Agroecosystems. 2005; 5: 57–66,

35. Nambiar EKS, Sands R. Effects of compaction and simulated root channels in the subsoil on root development, water uptake and growth of radiata pine. Tree Physiology. 1992; 10: 297–306 PMID: 14969986

36. Perrin AS, Fujisaki K, Petitjean C, Sarrazin M, Godet M, Garric B, et al. Conversion of forest to agriculture in Amazonia with the chop-and-mulch method: Does it improve the soil carbon stock? Agriculture, Ecosystems & Environ. 2014; 184: 101–114.

37. Portella CMR, Guimarães MF, Feller C, Fonseca ICB, Filho JT. Soil aggregation under different management systems. Rev. Bras. Ciência Solo. 2012; 36: 1868–1877

38. Tolk JA. Plant Available Soil Water. United States Department of Agriculture (USDA), Bushland, Texas, U.S.A. Encyclopedia of Water Science. doi: 10.1081/E-EWS 120010265 Marcel Dekker, Inc. 669–672. 2003. Available: http://www.cprl.ars.usda.gov/pdfs/Tolk-Plant%20Available%20Water-Ency%20Water%20Sci.pdf. Accessed 29 December 2014

39. Azam MG, Zoebisch MA, Wickramarachchi KS. Effects of cropping systems on selected soil structural properties and crops yields in the Lam phra phloeng watershed-northeast Thailand. J Agron. 2008; 7: 56–62

40. Amusan AA, Shitu AK, Makinde WO, Orewole O. Assessment of changes in selected soil properties under different land use in Obafemi Awolowo University Community, ILE-IFE, Nigeria. Electron J Environ Agric Food Chem. 2006; 5: 1178–1184.

41. Lambers H, Chapin FS III, Pons TL. Plant physiological ecology, second edition. Springer, New York. 2008. 634 p

42. He J, Li H, Rasaily RG, Wang Q, Cai G, Su Y, et al. Soil properties and crop yields after 11 years of no tillage farming in wheat-maize cropping system in North China Plain. Soil & Tillage Res. 2011; 113: 48–54

43. Gregory PJ. Plant roots: growth, activity and interactions with the soil. Blackwell Scientific Publishing, Oxford. 2006. 318pp.

44. Gil J, Zavala LM, Bellinfante N, Jordán A. Acidez y capacidad de intercambio catiónico en los suelos afectados por incendios. Métodos de determinación e interpretación de resultados. In: Cerdà A, Jordán A, editors. Actualización en métodos y técnicas para el estudio de los suelos afectados por incendios forestales. Càtedra de Divulgació de la Ciència, Universitat de València, FUEGORED, Valencia, Spain. 2010. pp. 327–348.

45. Granged AJP, Jordán A, Zavala LM, Muñoz-Rojas M, Mataix-Solera J. Short-term effects of experimental fire for a soil under eucalyptus forest (SE Australia). Geoderma. 2011; 167–168: 125–134.

46. Granged AJP, Zavala LM, Jordán A, Bárcenas-Moreno G. Post-fire evolution of soil properties and vegetation cover in a Mediterranean heathland after experimental burning: A 3-year study. Geoderma. 2011; 164: 85–94.

47. Certini G. Effects of fire on properties of forest soils: A review. Oecologia. 2005; 143:1–10. PMID: 15688212

48. Ekinci H. Effect of forest fire on some physical, chemical and biological properties of soil in Çanakkale, Turkey. International J of Agriculture & Biology. 2006; 8: 102–106.

49. Bell MC, Raczkowski CW. Soil property indices for assessing short-term changes in soil quality. Renewable Agriculture and Food Systems. 2008; 23: 70–79.

50. Dai Z, Liu Y, Wang X, Zhao D. Changes in pH, CEC and exchangeable acidity of some forest soils in southern China during the last 32–35 years. 1998 Water, Air, & Soil Pollution. 1998; 108: 377–390.

51. Boruvka L, Mladkova L, Drabek O. Factors controlling spatial distribution of soil acidification and Al forms in forest soils. J. Inorg Biochem. 2005; 99: 1796–806. PMID: 16095712

52. De Schrijver A, Mertens J, Geudens G, Staelens J, Campforts E, Luyssaert S, et al. Acidification of forested podzols in North Belgium during the period 1950–2000. Sci Total Environ. 2006; 361: 189–195. PMID: 16061272

53. Porębska G, Apolonia Ostrowska A, Borzyszkowski J. Changes in the soil sorption complex of forest soils in Poland over the past 27 years. Sci Total Environ. 2008; 399: 105–112. doi: 10.1016/j.scitotenv.2008.03.029 PMID: 18485449

54. Ololade IA, Ajayi IR, Gbadamosi AE, Mohammed OZ, Sunday AG. A study on effects of soil physico-chemical properties on cocoa production in Ondo state. Modern Applied Science. 2010; 4: 35–43.

55. Fageria NK, Baligar VC, Clark RB. Micronutrients in Crop Production. Advances in Agronomy. 2002; 77: 185–268

56. Altieri MA, Nicholls CI. Una base agroecológica para el diseño de sistemas diversificados en el trópico. Manejo Integrado de Plagas y Agroecología. 2004; 73:8–20.

57. Altieri MA, Nicholls CI. El potencial agroecológico de los sistemas agroforestales en América Latina. Leisa—Revista de Agroecología. 2011; 27: 32–35.

58. Pardini G, Gispert M, Dunjo G. Relative influence of wildfire on soil properties and erosion processes in different Mediterranean environments in NE Spain. Sci Total Environ. 2004; 328: 237–246. PMID: 15207587

59. Badía D, Martí C. Plant ash and heat intensity effects on chemical and physical properties of two contrasting soils. Arid Land Research and Management. 2003; 17: 23–41.

60. Seguel O, Baginsky C, Contreras A, Covarrubias JI, González C, Poblete L. Physical properties of a fine textured haplocambid after three years of organic matter amendments management. J Soil Sci and Plant Nutrition. 2013; 13: 690–705.

61. Wood GAR, Lass RA. Cocoa, Fourth Edition. Blackwell Science. Iowa State Univ. Press, Ames, IA. 2001. 75p

62. Alexis MA, Rasse DP, Rumpel C, Bardoux G, Péchot N, Schmalzer P, et al. Fire impact on C and N losses and charcoal production in a scrub oak ecosystem. Biogeochemistry. 2007; 82: 201–216.

63. Roscoe R, Buurman P, Velthorst EJ, Pereira JAA. Effects of fire on soil organic matter in a “cerrado sensu-stricto” from Southeast Brazil as revealed by changes in δ13C. Geoderma. 2000; 95: 141–160.

64. Johnson DW, Curtis PS. Effects of forest management on soil C and N storage: meta analysis. Forest Ecology and Management. 2001; 140: 227–238.

65. Czimczik CI, Schmidt MWI, Schulze ED. Effects of increasing fire frequency on black carbon and organic matter in Podzols of Siberian Scots pine forests. European J Soil Sci. 2005; 56: 417–428.

66. Brye KR. Soil physiochemical changes following 12 years of annual burning in a humid-subtropical tall-grass prairie: a hypothesis. Acta Oecologica, 2006; 30: 407–413.
67. Havlin JL, Beaton JD, Tisdale SL, Nelson WL. Soil fertility and fertilizers: An introduction to nutrient management, 7th Edition. Prentice Hall, Upper Saddle River, NJ. 2005
68. Are KS, Oluwatosin GA, Adeyolanu OD, Oke DO. Slash and burn effect on soil quality of an Alfisol: Soil physical properties. Soil and Tillage Research. 2009; 103: 4–10
69. Bot A, Benites J. The importance of soil organic matter: Key to drought-resistant soil and sustained food production. FAO Soils Bulletin 80. FAO, Rome. 2005. pp.71-78.
70. Magdoff F. Calidad y manejo del suelo. Capítulo 16. En: Altieri MA, editior. Agroecología: Bases científicas para una agricultura sustentable. Editorial Nordan-Comunidad, Montevideo. 1999
71. Gómez-Rey MX, Couto-Vázquez A, García-Marco S, González-Prieto SJ. Impact of fire and post-fire management techniques on soil chemical properties. Geoderma. 2013; 195–196: 155–164.
72. Juo ASR, Manu A. Chemical dynamics in slash-and-burn agriculture. Agriculture, Ecosystems & Environment. 1996; 58: 49–60.
73. Mendonca-Santos ML, Dos Santos HG, Coelho MR, Bernardi ACC, Machado PLOA, Manzatto CV, et al. Soil and land use in the Brazilian Amazon. In: Moreira FMS, Siqueira JO, Brussaard L, editors. Soil Biodiversity in Amazonian and Other Brazilian Ecosystems. CABI Publishing. London, UK. 2006. pp. 13–42.
74. Agoumé V, Birang AM. Impact of land-use systems on some physical and chemical soil propierties of an Oxisol in the Humid Forest Zone of Southern Cameroon. Tropicultura. 2009; 27: 15–20.
75. Thomaz EL, Antoneli V, Doerr SH. Effects of fire on the physicochemical properties of soil in a slash-and-burn agriculture. Catena. 2014; 122: 209–215
76. Wang QJ, Bai YH, Gao HW, He J, Chen H, Chesney RC, et al. Soil chemical properties and microbial biomass after 16 years of no-tillage farming on the Loess Plateau, China. Geoderma. 2008; 144: 502–508.
77. Villegas R. Descomposición de las hojas del cacao y de seis especies arbóreas, solas y en mezcla en Alto Beni, Bolivia (Decomposition of cocoa leaves and six arboreal shade species, alone and mixed, in Alto Beni, Bolivia). Tesis Mag. Sci. CATIE, Turrialba, Costa Rica. 2008. 82 p. Available: http://orton.catie.ac.cr/repdoc/A1823E/A1823E.PDF
78. Close DC, Davidson NJ, Swanborough PW, Corkrey R. Does low-intensity surface fire increase water- and nutrient-availability to overstorey Eucalyptus gomphocephala?. Plant Soil. 2011; 349: 203–214
79. García-Marco S, González-Prieto S. Short- and medium-term effects of fire and fire-fighting chemicals on soil micronutrient availability. The Science of the Total Environment. 2008; 407 (1), 297–303. doi: 10.1016/j.scitotenv.2008.08.021 PMID: 18805571
80. Danoff LE, Elmer WH, Huber DM. Mineral nutrition and plant disease. The American Phytopathological Society, St. Paul, Minnesota, USA. 2007. 278p.
81. Alegre JC, Cassel DK, Bandy DE. Effect of land clearing method on chemical properties of an Ultisol in the Amazon. Soil Sci. Soc. Am. J. 1988; 52:1283–1288
82. Couto-Vázquez A, González-Prieto SJ. Short- and medium-term effects of three fire fighting chemicals on the properties of a burnt soil. Sci Total Environ. 2006; 371: 353–361. PMID: 17011025
83. Theodoro VCA; Alvarenga MIN, Guimarães RJ, Souza CAS. Chemical changes of a soil under different management forms of coffee plantation. Revista Brasileira de Ciência do Sólo. 2003; 27(6):1039–1047.
84. Moussa-Machraoui SB, Errouissi F, Ben-Hammouda M, Nouira S. Comparative effects of conventional and no-tillage management on some soil properties under Mediterranean semi-arid conditions in north-western Tunisia. Soil and Tillage Research. 2010; 106: 247–253.
85. Barreto AC, Soriano LFH, Galvão dos SFMB, Reis de AQ, Freire FJ. Chemical and physical characteristics in soil under forest, agroforest system and pasture. Revista Caatinga (Mossoró,Brasil), 2006; 19: 415–425.
86. Dinesha R, Srinivasan V, Hamza S, Parthasarathy VA, Aipe KC. Physico-chemical, biochemical and microbial properties of the rhizospheric soils of tree species used as supports for black pepper cultivation in the humid tropics. Geoderma. 2010; 158: 252–258.
87. Phillips RP, Fahey TJ. Tree species and mycorrhizal associations influence the magnitude of rhizosphere effects. Ecology. 2006 87: 1302–1313. PMID: 16761608
88. Radersma S, Grierson PF. Phosphorus mobilization in agroforestry: organic anions, phosphatase activity and phosphorus fractions in the rhizosphere. Plant Soil. 2004; 259: 209–219.
89. McGrath DA, Duryea ML, Cropper WP. Soil phosphorus availability and fine root proliferation in Amazonian agroforests 6 years following conversion. Agric Ecosystems & Environ. 2001; 83: 271–284.

90. Alfaia SS, Ribeiro GA, Nobre AD, Luizão RC, Luizão FJ. Evaluation of soil fertility in smallholder agroforestry systems and pastures in western Amazonia. Agric Ecosystem Environ. 2004; 102: 409–414.
91. Phillips RP, Fahey TJ. The influence of soil fertility on rhizosphere effects in northern hardwood forest soils. Soil Sci. Soc. Am. J. 2008; 72: 453–461
92. Russell AE, Raich JW, Valverde-Barrantes OJ, Fisher RF. Tree species effects on soil properties in experimental plantations in tropical moist forest. Soil Sci. Soc. Am. J. 2007; 71: 1389–1397.
93. Jobbágy EG, Jackson RB. The uplift of soil nutrients by plants: biogeochemical consequences across scales. Ecol. 2004; 85: 2380–2389.
94. Ayres E, Steltzer H, Berg S, Wallenstein MD, Simmons BL, Wall DH. Tree species traits influence soil physical, chemical, and biological properties in high elevation forests. PLoS ONE. 2009; 4: 5964.
